# LukS-PV inhibits hepatocellular carcinoma cells migration by downregulating HDAC6 expression

**DOI:** 10.1186/s12885-022-09680-4

**Published:** 2022-06-08

**Authors:** Xuexue Xu, Pengsheng Ding, Lan Shi, Gang Wu, Xiaoling Ma

**Affiliations:** The First Affiliated Hospital of USTC, Division of Life Sciences and Medicine, University of Science and Technology of China, Hefei, Anhui China

**Keywords:** Hepatocellular carcinoma, LukS-PV, Migration, HDAC6, α-tubulin

## Abstract

**Background:**

Hepatocellular carcinoma (HCC) is a clinically common malignant tumor worldwide. LukS-PV is the S component of Panton-Valentine leukocidin secreted by *Staphylococcus aureus*, which has shown anti-cancer activity. Based on previous findings, this study investigated the effects of LukS-PV on HCC migration and the potential molecular mechanisms involving acetylation pathways.

**Methods:**

After treating HCC cells with different concentrations of LukS-PV, we used scratch assays to determine the mobility of the cancer cells. Western blots were used to determine the expression levels of migration-related proteins. Quantitative proteomic sequencing was used to evaluate proteomic changes in target proteins. Immunoprecipitation and liquid chromatography coupled with tandem mass spectrometry analyses were used to validate the binding of related target proteins.

**Results:**

LukS-PV inhibited HCC cell migration in a concentration-dependent manner. In addition, LukS-PV attenuated the expression of histone deacetylase (HDAC)6, which is highly expressed in HCC cells. Further studies showed that LukS-PV increased the acetylation level of α-tubulin by down-regulating HDAC6, which resulted in the inhibition of HCC cell migration.

**Conclusion:**

Taken together, our data revealed a vital role of LukS-PV in suppressing HCC cell migration by down-regulating HDAC6 and increasing the acetylation level of α-tubulin.

**Supplementary Information:**

The online version contains supplementary material available at 10.1186/s12885-022-09680-4.

## Background

Hepatocellular carcinoma (HCC) is the sixth most common and the fourth most deadly cancer worldwide [1]. The high mortality rate of HCC is closely related to its ability to undergo metastasis [2]. Currently, the main treatments for HCC include surgical resection, radiotherapy, and chemotherapy [3]; however, the high postoperative recurrence and low 5-year survival rates remain problematic [4]. Therefore, it is necessary to understand the underlying mechanisms that regulate metastasis of HCC. Furthermore, finding new molecular targets with therapeutic potential is critical for HCC prevention and improving treatment.

Bacterial toxins have specificity and cytotoxicity against different cancer cells and have become a new focus for the development of novel antitumor drugs. For example, EGFuPA-toxin is a bispecific ligand-targeted toxin consisting of deimmunized *Pseudomonas* exotoxin A that exhibits anticancer activity against sarcoma and canine hemangiosarcoma [5]. Cytolethal distending toxin is a secreted tripartite genotoxin produced by many pathogenic gram-negative bacteria and exhibits anticancer activity against lung cancer [6]. Cytotoxic necrotizing factor 1 produced by pathogenic *Escherichia coli* strains is cytotoxic against gliomas [7] and has been shown to promote inflammation, stimulate host immunity, and function as a potent immunoadjuvant [8]. Panton-Valentine Leukocidin (PVL) is a perforating toxin secreted by *Staphylococcus aureus*, which was first discovered by Panton and Valentine and proved to be associated with skin and soft tissue infection in 1932 [9]. PVL contained two components, LukS-PV and LukF-PV [10]. LukS-PV, as the initial factor of the interaction between PVL and target cells, identifies and binds to the C5aR of the target cell membrane, then binds to LukF-PV to form a dimer. The dimer is further assembled to form an octamer complex pore structure, which is inserted into the cellular membrane to form a transmembrane pore perpendicular to the membrane plane that induces cell apoptosis [11, 12]. In vitro and in vivo experiments have shown that LukS-PV alone did not induce cell perforation, but inhibited the proliferation of human acute myeloid leukemia cells [13, 14]. Furthermore, LukS-PV inhibited HCC progression by downregulating histone deacetylase (HDAC)2 expression [15].

Histone acetylation and deacetylation are regulated by histone acetyltransferases (HATs) and HDACs, respectively, and these modifications play critical roles in gene expression and transcription [16]. The role of abnormally high HDAC expression in promoting tumorigenesis and cancer development has been widely recognized [17, 18]. HDAC6, a member of the HDAC Class II family, is highly expressed in breast [19], lung [20], bladder cancers [21], and HCC [22]. Several studies have shown that HDAC6 can participate in the process of carcinogenesis through various pathways, including oncogenic cellular transformation and cancer cell migration and invasion [23–25]. Thus, HDAC6 is considered to be a therapeutic target for some tumors.

Our previous study demonstrated that LukS-PV inhibited HCC cell migration by interfering with PI3K-AKT signaling [26]. However, it remains unknown whether LukS-PV can inhibit HCC cell migration through acetylation pathways. In this study, we demonstrated that LukS-PV inhibited HCC cell migration by down-regulating HDAC6 and increasing the acetylation level of α-tubulin. These data suggest that LukS-PV plays an important role in inhibiting HCC migration and may have potential as a therapeutic drug.

## Methods

### Cell culture

The human HCC cell lines HepG2, Bel-7402, Hep3B, Huh-7, normal human liver cell line LO2, and human embryonic kidney cell (HEK293T) were purchased from Shanghai Cell Bank (Chinese Academy of Sciences, Shanghai, China). Bel-7402 and LO2 cells were cultured in RPMI-1640 medium (GIBCO, USA) supplemented with 10% fetal bovine serum, 100 U/mL penicillin, and 100 mg/mL streptomycin. HEK293T, HepG2, Hep3B, and Huh-7 cells were cultured in DMEM medium (GIBCO, USA) containing 10% fetal bovine serum, 100 U/mL penicillin, and 100 mg/mL streptomycin. Cells were grown as adherent monolayers in 25 cm^2^ culture flasks and cultured at 37 °C with 5% CO2 in a humidified incubator.

### Production and purification of recombinant LukS-PV

The LukS-PV sequence was amplified from PVL-positive *Staphylococcus aureus* isolates by polymerase chain reaction (PCR) using the following primers: LukS-FW, 5ʹ-acgcGGATCCGAATCTAAAGCTGATAACAATATTGAGAATATTG-3ʹ; LukS-RV, 5ʹ-accgCTCGAGTCAATTATGTCCTTTCACTTTAATTTCATGAG-3ʹ. Recombinant LukS-PV was generated as described previously [27]. Purification of recombinant LukS-PV was performed with the His-Bind® Purification Kit (Millipore, USA) in accordance with the manufacturer's instructions.

### Western blot assay

Cells were lysed in RIPA lysis buffer containing proteinase inhibitors at 4 °C for 20 to 30 min. Protein was quantified using a BCA Kit (Beyotime, China). Protein samples were boiled in 5 × loading buffer, and equal amounts of proteins were separated on 10% SDS-PAGE gels and transferred to nitrocellulose membranes. The membranes were blocked with 5% skim milk for 1.5 h and incubated with a primary antibody overnight at 4 °C, the blots were cut prior to hybridization with respective antibodies. The primary antibodies used were as follows: anti-HDAC6, anti-α-tubulin and anti-Acetyl-α-tubulin (Cell Signaling Technology, USA); anti-matrix metallopeptidase MMP2, anti-MMP9, and anti-GAPDH (Abclonal, China). The corresponding horseradish peroxidase-conjugated secondary antibodies were used following primary antibody incubation.

### Transfection

HDAC6 siRNA and the corresponding negative control were purchased from General Biotechnology (Anhui, China). HCC cells were transfected with siRNA at a final concentration of 50 nmol/L using Lipofectamine 2000 (Invitrogen, USA). Transfection efficiency was assessed by western blot. The siRNA sequences for transfection were as follows: negative control, sense: 5ʹ-UUC UCCGAA GGU GUC ACG UTT-3ʹ; antisense: 5ʹ-ACG UGA CAC GUU CGG AGA ATT-3ʹ); and HDAC6, sense: 5ʹ-GCA AAU ACU AUG CUG UCA ATT-3ʹ; antisense: 5ʹ-UUG ACA GCA UAG UAU UUG CTT-3ʹ.

### Lentiviral transduction

To establish stable cell lines overexpressing HDAC6, HDAC6 cDNA with a Flag-tag at the C-terminus was cloned into the pCDH-CMV-MCS-EF1-Puro vector. The recombinant vector was used to package pseudoviral particles in HEK293T cells by co-transfection with the two packaging plasmids psPAX2 and pMD2.G. The particles were harvested after 24 h and were used to infect Hep3B and Huh-7 cells. Stable pools of infected cells were selected with 2.5 μg/mL puromycin. After 96 h, the stable expression of HDAC6 was verified by western blot. The established Hep3B and Huh-7 cells were maintained in a growth medium containing 1 μg/mL puromycin.

### Cell scratch assay

The cells were inoculated into six-well plates at a density of 90% and cultured overnight in an incubator. A scratch perpendicular to the cell layer was created using a 10 μL pipette tip. Subsequently, the cells were rinsed twice with PBS to remove loose cells, and fresh serum-free medium was added to continue the culture. Images were captured at 0 h and 48 h after the scratch assay with an inverted microscope to assess migration distance at the scratch region. The percentage of relative migration = (gap width at 0 h—gap width at 48 h)/(gap width at 0 h) × 100%. Three duplicated wells were set in each well and the experiment was repeated three times.

### Quantitative proteomics sequencing

HepG2 cells were treated with 0.5 μmol/L LukS-PV or equivalent amount of PBS (control) for 24 h, then cells were sonicated three times in lysis buffer on ice using a high-intensity ultrasonic processor (Scientz, China). After collecting the supernatant, the protein concentrations were determined with a BCA kit as described above. The proteins were reduced with 5 mM dithiothreitol and alkylated with 11 mM iodoacetamide. The urea in the lysis buffer was diluted to less than 2 M by adding 100 mM tetraethyl ammonium bromide (TEAB). Finally, trypsin was added for overnight protein digestion.

After trypsin digestion, the peptides were reconstituted in 0.5 M TEAB and labeled using the TMT kit/iTRAQ kit in accordance with the manufacturer's protocol. The polypeptides were isolated and purified using an Agilent 300Extend C18 column by high pH, reverse-phase high performance liquid chromatography. The peptides were added to a nanoelectrospray ionization source followed by tandem mass spectrometry (MS/MS) using a Q Exactive Plus mass spectrometer (Thermo Fisher Scientific, USA) coupled to an ultraperformance liquid chromatography system. The resulting MS/MS data were processed using the MaxQuant proteomics software package and search engine (V.1.5.2.8).

### Immunopurification and mass spectrometry

The Huh-7 and Hep3B cell lines with stable expression of HDAC6 were lysed in RIPA lysis buffer and centrifuged at 10,000 g for 10 min at 4 °C. IgG and the anti-HDAC6 primary antibody were added to the lysates. After overnight incubation at 4 °C, 30 μL protein A/G agarose beads were added to the lysates and incubated at 4 °C for 3 h. The beads were washed 3 times with lysis buffer followed by boiling at 100 °C for 10 min in 2 × SDS loading buffer. Proteins in the supernatant were analyzed by western blot. Additionally, the proteins in the supernatant from the Huh-7 cell line expressing HDAC6 were separated by SDS-PAGE, and the gel was stained with Coomassie brilliant blue. Whole lane after staining the gel was removed and sent to General Biotechnology (Anhui, China) for mass spectrometry analysis.

### Site-directed mutation of the α-Tubulin locus

Total RNA was extracted from Huh-7 cells and α-tubulin mRNA was amplified by RT-PCR. The recovered DNA was digested with the appropriate enzymes and cloned into the eukaryotic expression vector pcDNA3-1(-). Using this wild-type α-tubulin expression plasmid as the template, lysine 40 residue in the α-tubulin N-terminal domain was mutated to glutamine residue as acetylation-mimicking mutant (K40Q) using the QuickMutation Plus Site-Directed Mutagenesis Kit (Beyotime, China). Additionally, lysine 40 residue in α-tubulin was mutated to arginine residue to generate an acetylation-resistant mutant (K40R). After confirmational sequencing, these mutant plasmids were used in subsequent experiments.

### Statistical analysis

All performances were at least triple. Data were presented as mean ± SD. The statistical analyses were performed using the statistical software package GraphPad Prism 8 (Graphpad Software, USA). The unpaired Student's t-test was used to analyze the differences between two groups, and differences between multiple groups were analyzed by ANOVA. *p < 0.05, **p < 0.01, or ***p < 0.001 was considered statistically significant.

## Results

### LukS-PV inhibited the migration of HCC cells

**Fig. 1:**
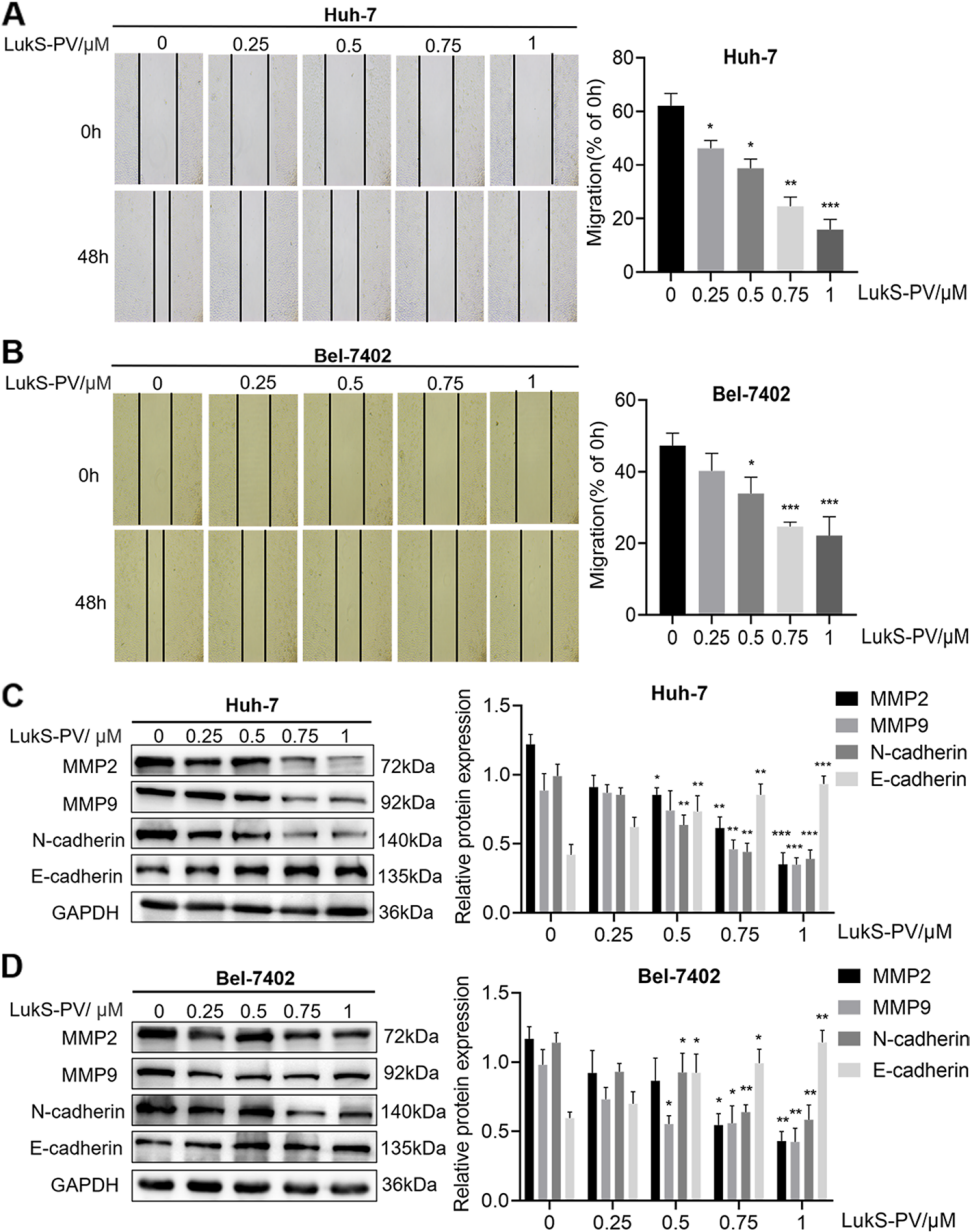
LukS-PV inhibited the migration of HCC cells. **A**-**B** The mobility of HCC cells treated with different concentrations of LukS-PV was examined by scratch assays, and the percentage of relative migration was calculated for Huh-7 and Bel-7402 cells. **C**-**D** The protein expression levels of MMP2, MMP9, N-cadherin and E-cadherin were detected by western blot in HCC cells after treatment with different concentrations of LukS-PV. Relative band intensities (compared to GAPDH) were measured using Image J. * *P* < 0.05, ** *P* < 0.01, or *** *P* < 0.001 compared with the control cells

To verify that LukS-PV inhibited the migration of HCC cells, we treated Huh-7 and Bel-7402 cells with different concentrations of LukS-PV for 48 h, and scratch assays were used to detect the migration rates of these cells. Compared with migration in the control group, the migration capacity of Huh-7 and Bel-7402 cells was reduced after 48 h treatment with LukS-PV. Moreover, the reduction in migration was positively correlated with the concentration of LukS-PV (Fig. 1A-B). To further investigate the molecular mechanism, we examined migration-associated proteins in the HCC cells. As presented in Fig. 1C and D, LukS-PV treatment decreased matrix metallopeptidase (MMP) 2, MMP9, N-cadherin expression, and increased the expression of E-cadherin. Taken together, these results indicated that LukS-PV inhibited the migration of HCC cells.

### LukS-PV downregulated the high expression of HDAC6 in HCC cells

**Fig. 2:**
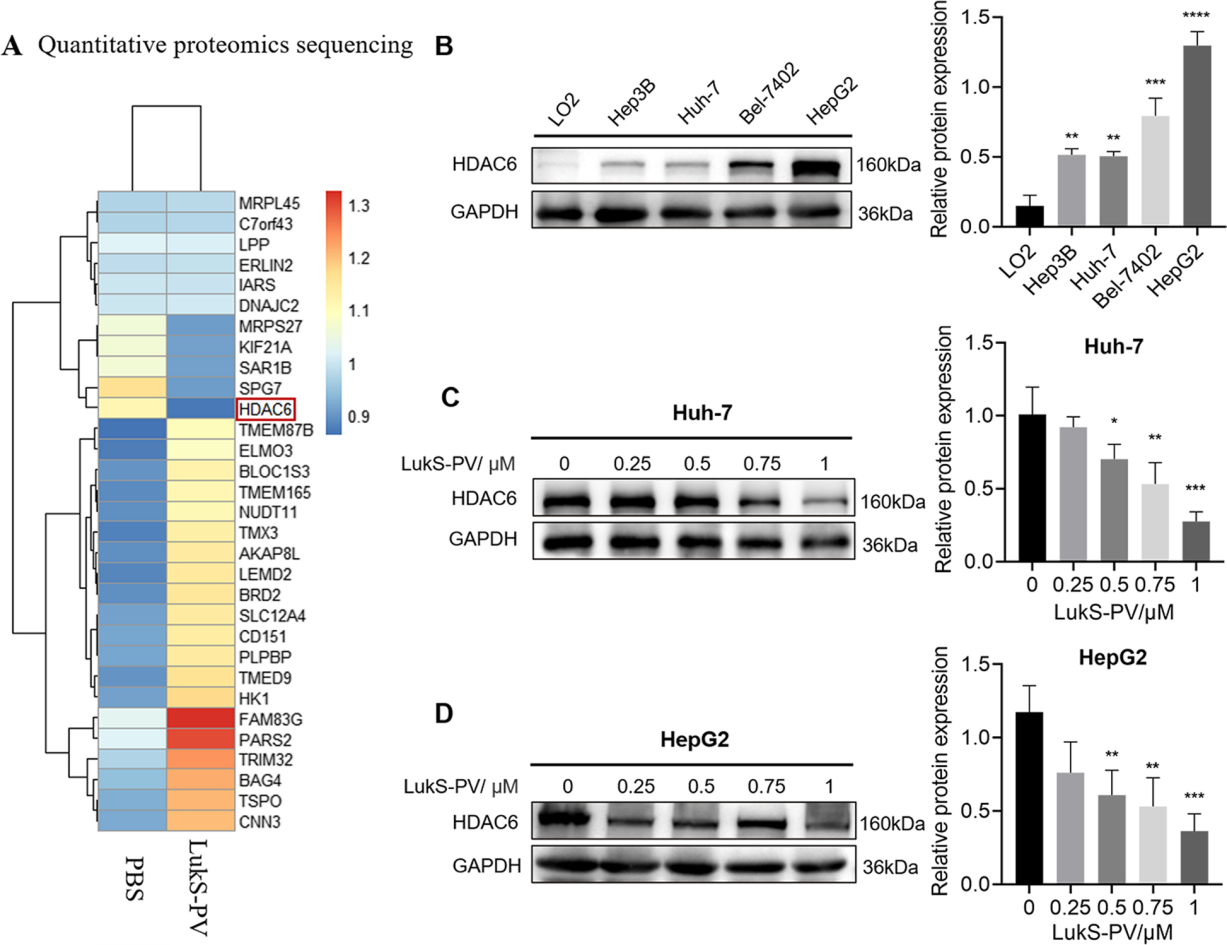
LukS-PV downregulated the high expression of HDAC6 in HCC cells. **A** Quantitative proteomics sequencing was used to detect changes in HDAC6 expression levels in HepG2 cells treated with LukS-PV or PBS. **B** The protein expression of HDAC6 was detected by Western blot in LO2, Hep3B, Huh-7, Bel-7402, and HepG2 cells. Relative band intensities (compared to GAPDH) were measured using Image J. **C**-**D** The expression levels of HDAC6 protein were determined in Huh-7 and HepG2 cells after treatment with different concentrations of LukS-PV. Relative band intensities (compared to GAPDH) were measured using Image J. * *P* < 0.05, ** *P* < 0.01, or *** *P* < 0.001 compared with the control cells

To understand the potential mechanism by which LukS-PV inhibited HCC cell migration, we used quantitative proteomics sequencing to determine proteomic changes in HepG2 cells after PBS or LukS-PV treatment (Supplementary Table S1). Interestingly, we found that the deacetylase HDAC6 was down-regulated in the LukS-PV treatment group compared with the PBS control group (Fig. 2A). To further investigate the role of HDAC6 in HCC, we evaluated the expression levels in the normal liver cell line LO2 and liver cancer cell lines Huh-7, Hep3B, Bel-7402, and HepG2 and demonstrated that HDAC6 was indeed highly expressed in HCC cells. The expression of HDAC6 in Bel-7402 and HepG2 cells was relatively high in comparison with the expression of HDAC6 in Huh-7 and Hep3B cells (Fig. 2B). In addition, we verified that LukS-PV down-regulated HDAC6 in the HCC cell lines Huh-7 and HepG2 by western blot (Fig. 2C-D). Together, these data revealed that LukS-PV treatment reduced HDAC6 expression in HCC cells.

### LukS-PV inhibited HCC cell migration by downregulating HDAC6

**Fig. 3:**
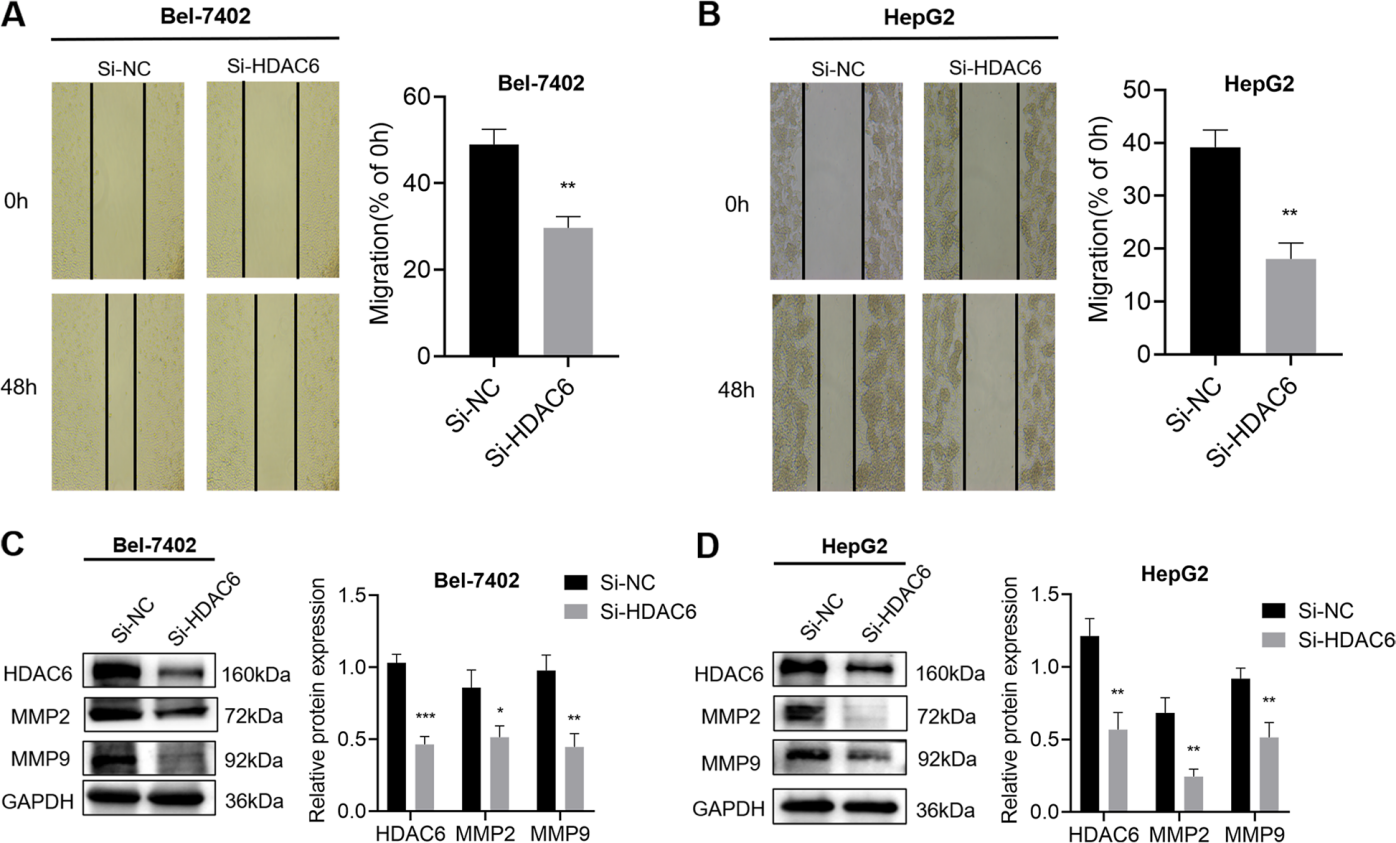
Knockdown HDAC6 inhibited HCC cells migration. **A**-**B** The mobility of Bel-7402 and HepG2 cells were examined by scratch assays after knocking down HDAC6 with siRNA, and the percentage of relative migration was calculated. **C**-**D** The protein expression levels of HDAC6, MMP2 and MMP9 were determined in Bel-7402 and HepG2 cells after knocking down HDAC6. Relative band intensities (compared to GAPDH) were measured using Image J. * *P* < 0.05, ** *P* < 0.01, or *** *P* < 0.001 compared with the control cells

**Fig. 4:**
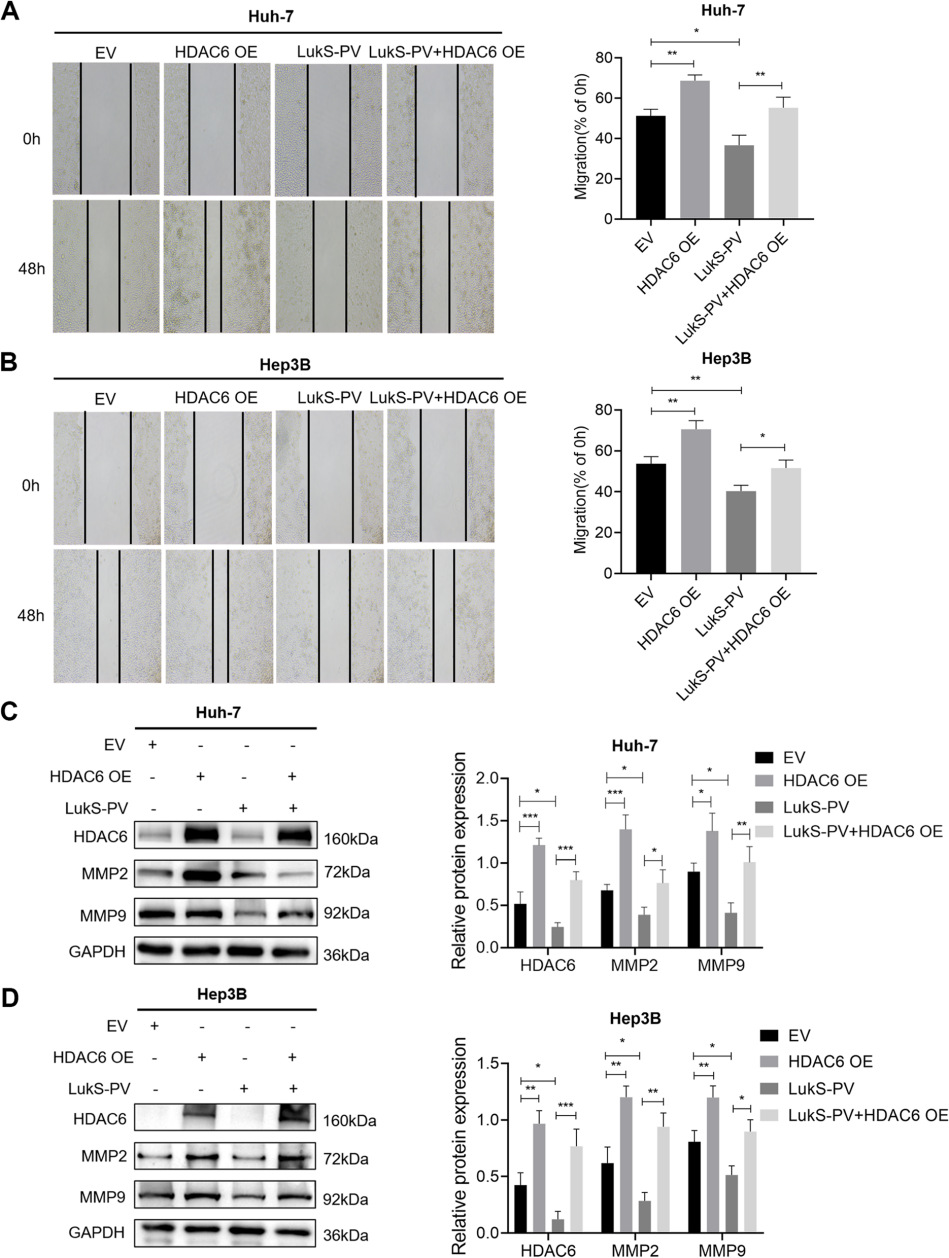
LukS-PV inhibited HCC cells migration by downregulating HDAC6. **A**-**B** Huh-7 and Hep3B cells were infected with EV (empty vector) or the vector overexpressing HDAC6 (HDAC6 OE) and treated with LukS-PV. The mobility of these cells was examined by scratch assays, and the percentage of relative migration was calculated. **C**-**D** The protein expression levels of HDAC6, MMP2 and MMP9 were detected by western blot. Relative band intensities (compared to GAPDH) were measured using Image J. * *P* < 0.05, ** *P* < 0.01, or *** *P* < 0.001

To clarify the role of HDAC6 in HCC cell migration, we knocked down HDAC6 expression in HepG2 and Bel-7402 cells, which were the cell lines with relatively high HDAC6 expression. SiRNA-mediated knockdown of HDAC6 resulted in a significant reduction in the migration of HepG2 and Bel-7402 cells (Fig. 3A-B). The expression of HDAC6, MMP2 and MMP9 were significantly down-regulated in Bel-7402 and HepG2 cells compared with the control cells (Fig. 3C-D). Huh-7 and Hep3B cells were selected to perform overexpression and rescue experiments, and scratch experiments showed that HDAC6 overexpression significantly increased the migration ability of these cells (Fig. 4A-D). In addition, the ectopic expression of HDAC6 significantly decreased the inhibitory effect of LukS-PV on Huh-7 and Hep3B cell migration compared with LukS-PV-treated vector-only control cells (Fig. 4A-D). These results suggested that LukS-PV inhibited HCC cell migration by down-regulating HDAC6.

### LukS-PV inhibits HCC cell migration by increasing α-Tubulin acetylation through HDAC6 downregulation

**Fig. 5:**
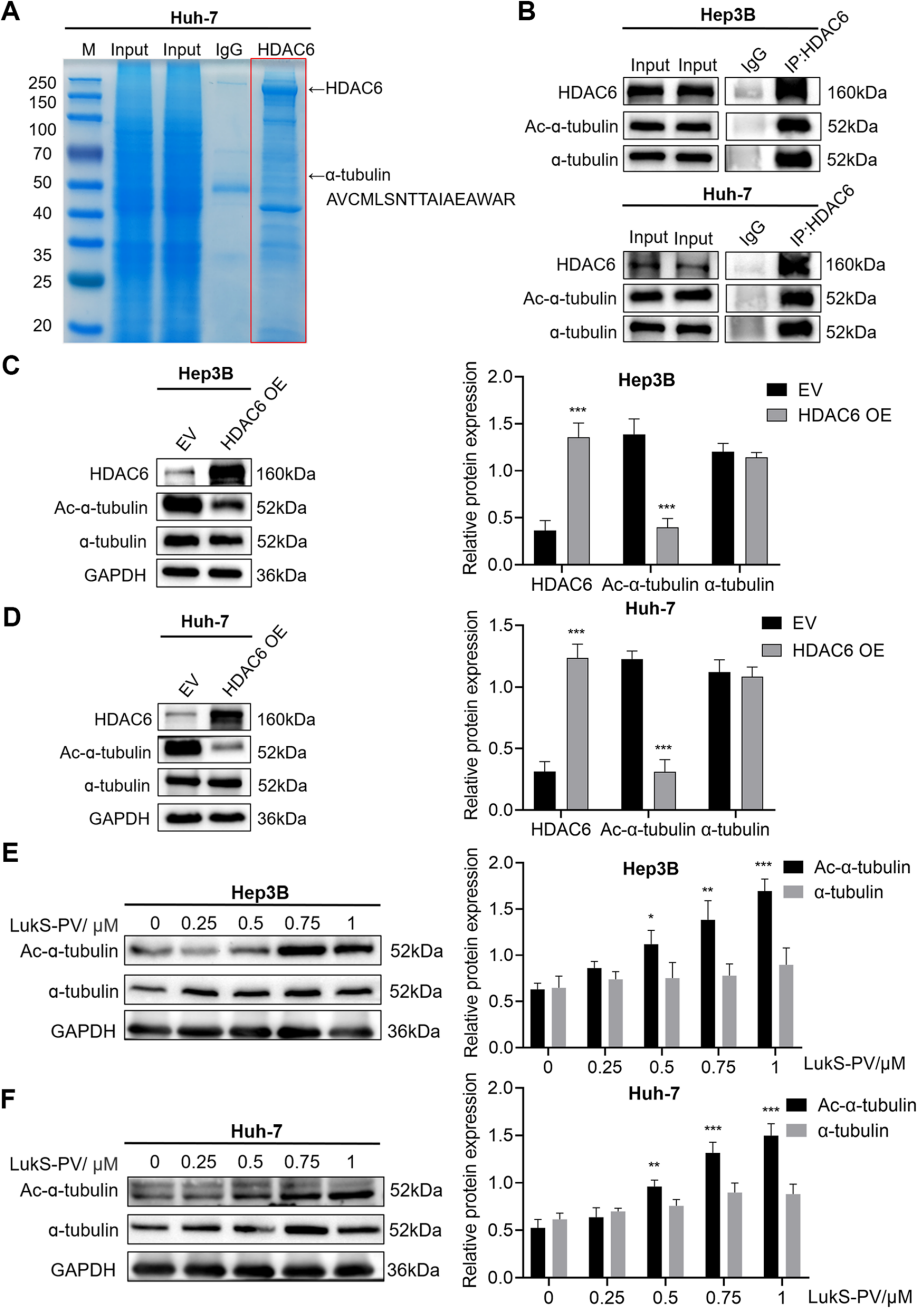
LukS-PV increasing α-tubulin acetylation through HDAC6 downregulation. **A** A whole-cell extract from Huh-7 cells stably expressing HDAC6 was subjected to affinity purification with protein A/G beads. The purified protein complex was resolved by SDS–polyacrylamide gel electrophoresis followed by staining with Coomassie brilliant blue. The bands were retrieved and analyzed by mass spectrometry. The peptide (AVCMLSNTTAIAEAWAR) showing in this panel is the α-tubulin sequence detected by mass spectrometry. **B** The whole-cell lysate and sample from the co-immunoprecipitation with anti-HDAC6 antibody in HCC cells were verified by western blot. **C-D** The indicated proteins were verified by western blot after transfection of HCC cells with the HDAC6 plasmid. Relative band intensities (compared to GAPDH) were measured using Image J. **E**–**F** The expression levels of α-tubulin and its acetylated form were determined in HCC cells after treatment with different concentrations of LukS-PV. Relative band intensities (compared to GAPDH) were measured using Image J. * *P* < 0.05, ** *P* < 0.01, or *** *P* < 0.001

**Fig. 6:**
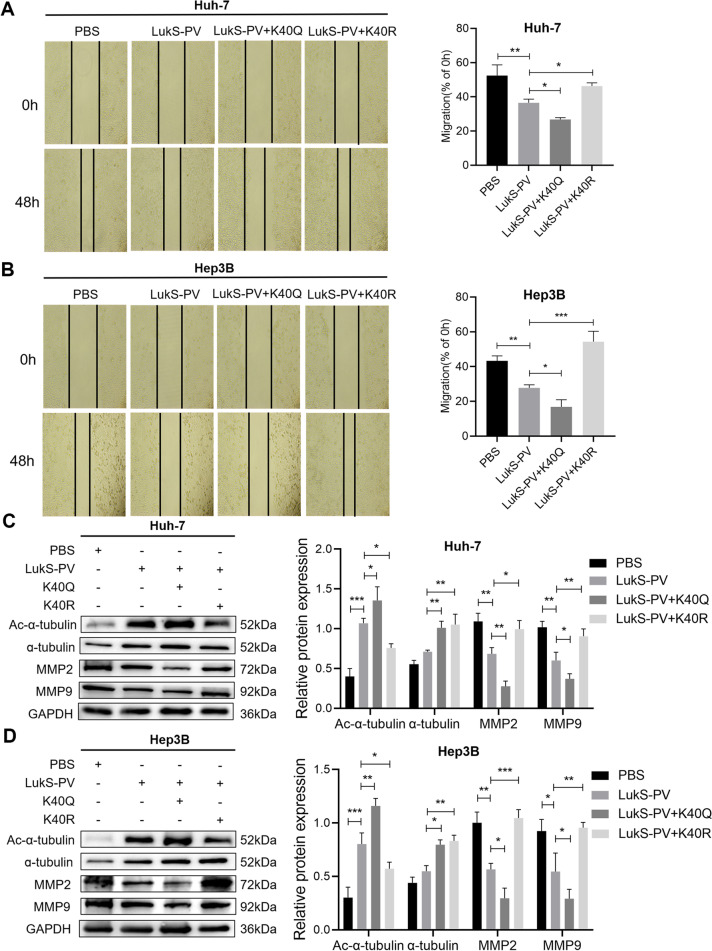
LukS-PV inhibited HCC cells migration by increasing α-tubulin acetylation. **A-B** After Huh-7 and Hep3B cells were transfected with the α-tubulin K40Q and K40R mutant plasmids, they were treated with PBS or LukS-PV, and the mobility of cells was examined by scratch assays. The percentage of relative migration was calculated. **C-D** The protein expression levels of Ac-α-tubulin, α-tubulin, MMP2 and MMP9 were detected by western blot. Relative band intensities (compared to GAPDH) were measured using Image J. * P < 0.05, ** P < 0.01, or *** P < 0.001

**Fig. 7:**
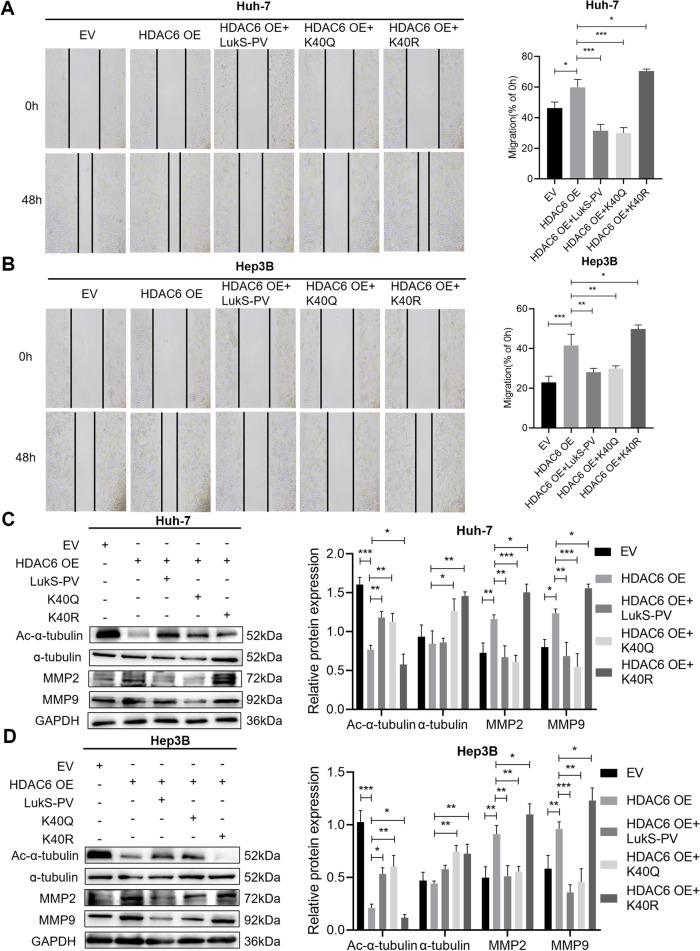
LukS-PV inhibited HCC cells migration by increasing α-tubulin acetylation through HDAC6 downregulation. **A–B** Huh-7 and Hep3B cells containing EV (empty vector) or overexpressing HDAC6 (HDAC6 OE) were transfected with either the α-tubulin K40Q or K40R mutant plasmids and treated with LukS-PV. The mobility of cells was examined by scratch assays. The percentage of relative migration was calculated. **C-D** The protein expression levels of Ac-α-tubulin, α-tubulin, MMP2 and MMP9 were detected by western blot. Relative band intensities (compared to GAPDH) were measured using Image J. * P < 0.05, ** P < 0.01, or *** P < 0.001

To further investigate the molecular mechanisms by which HDAC6 regulated HCC migration, immunoprecipitation and liquid chromatography-MS/MS analyses were used to examine potential HDAC6-interacting proteins in Huh-7 cells stably expressing HDAC6 (Supplementary Table S2). As shown in Fig. 5A, we demonstrated that α-tubulin is associated with HDAC6, and it may act as a downstream target of HDAC6 to affect the migration of HCC cells. Next, we validated the relationship between α-tubulin and HDAC6 by immunoprecipitation (Fig. 5B), and found that HDAC6 negatively regulated the acetylation level of α-tubulin (Fig. 5C-D). Therefore, we hypothesized that LukS-PV might inhibit HCC cell migration by down-regulating HDAC6 and subsequently increasing the acetylation level of α-tubulin. To verify this hypothesis, western blot analyses were performed and revealed that the LukS-PV treatment group had a significant increase in acetylated α-tubulin when compared with the control group (Fig. 5E-F). In addition, the lysine 40 (K40) residue of α-tubulin was identified as the major acetylation site [28]. Furthermore, Li et al. showed that overexpression of tubulin K40Q to mimic acetylated α-tubulin could reduce the migratory and morphological defects caused by MEC-17 deficiency in cortical projection neurons [29]. Therefore, on the basis of the above studies, we selected the highly conserved K40 locus to construct K40Q acetylation-mimicking mutant and K40R acetylation-resistant mutant to further study whether the acetylation level of K40 affects the migration of HCC cells. Subsequently, scratch assays and western blots were used to determine migration rates, migration-related proteins, and acetylation levels of α-tubulin in HCC cells. Rescue experiments showed that the K40Q α-tubulin mutation enhanced the inhibitory effect of LukS-PV on the migration of HCC cells, while the K40R mutation reversed the inhibitory effect of LukS-PV on migration (Fig. 6A-D). Furthermore, the K40Q mutation reduced HDAC6-mediated enhancement of HCC cell migration, while the K40R mutation increased HDAC6-mediated migration in these cells (Fig. 7A-D). Interestingly, the acetylation level of α-tubulin were increased when cells were treated with K40Q. On the one hand, it may be that the acetylated antibody can still recognize K40Q with a similar structure to some extent. On the other hand, there may be other signaling pathways involved in regulating the acetylation level of α-tubulin, which needs to be paid attention to in future experiments.

In summary, these results demonstrated that LukS-PV inhibited the migration of HCC cells by down-regulating HDAC6, which resulted in an increased level of acetylated α-tubulin.

## Discussion

Hepatocellular carcinoma (HCC) is one of the most common solid malignancies and a leading cause of cancer-related death worldwide [30]. The survival of patients with HCC remains poor despite recent advances in the diagnosis and treatment over the past decades [1]. In recent years, bacterial toxins have attracted significant interest as potential tumor therapeutics [5]. In previous studies, we found that the bacterial toxin LukS-PV inhibited the proliferation of leukemia and HCC cells and induced cell cycle arrest and apoptosis [14, 15]. In this study, we demonstrated that LukS-PV inhibited the migration of HCC cells by regulating acetylation. To investigate the role of LukS-PV in HCC migration, HCC cell lines Huh-7 and Bel-7402 were treated with different concentrations of LukS-PV. Compared with migration in the control group, LukS-PV inhibited HCC cell migration in a concentration-dependent manner.

Acetylation has been shown to play an important role in the genesis and progression of tumors. Therefore, we searched for relevant targets that regulated acetylation using quantitative proteomic sequencing. HDAC6 caught our attention because it was significantly down-regulated in the LukS-PV treated group of cells. HDACs are often abnormally overexpressed in various tumors. Inhibition of HDAC can reestablish intracellular acetylation-deacetylation homeostasis and reverse the initiation and development of tumors [31]. Given that histone modification modulates chromatin structure and gene expression, it is not surprising that abnormal alterations in histone acetylation are associated with cancer development [31, 32]. In addition, it has been reported that HDAC6 can regulate the acetylation of α-tubulin and, thus, participate in tumor development [33].

α-Tubulin is an important component of microtubules. Reversible acetylation of α-tubulin can modulate the stability and dynamic activity of microtubules and subsequently regulate microtubule-mediated processes, such as cell shape maintenance, mitosis, migration, intracellular trafficking, and determination of cell fate, i.e., survival or apoptosis [34, 35]. It has been reported that the level of α-tubulin acetylation is crucial for determining the adhesive and migratory ability of cancer cells [36]. For example, in lung cancer cells, an increase in α-tubulin acetylation increases cell adhesion and inhibits cell migration, invasion, and tumor metastasis [37]. In this study, we first verified the binding of HDAC6 to α-tubulin and demonstrated that HDAC6 negatively regulated the acetylation level of α-tubulin. In addition, we demonstrated that LukS-PV increased the acetylation level of α-tubulin in a concentration-dependent manner. Further experiments demonstrated that LukS-PV inhibited HCC cell migration by downregulating HDAC6 and increasing the acetylation level of α-tubulin.

There are some limitations in this study. First, we demonstrated that HDAC6 affected the migration of HCC cells by regulating the acetylation level of α-tubulin. However, further studies will be required to discover other migration-related target proteins that are downstream targets of HDAC6 based on the immunopurification and mass spectrometry results. Second, we used cell lines to demonstrate that LukS-PV can inhibit HCC cell migration in vitro. In future experiments, other models, such as nude mice xenografted with patient-derived tumors, will be important to verify whether LukS-PV can inhibit HCC cell migration in vivo.

## Conclusion

In conclusion, this study demonstrated that LukS-PV increased the acetylation level of α-tubulin via HDAC6, thereby inhibiting the migration of HCC cells, indicating that LukS-PV may have potential as a promising candidate for the treatment of HCC.

## Supplementary Information

**Additional file 1: Supplementary Table S1.** Proteomic changes in HepG2 cells after PBS or LukS-PV treatment were determined with quantitative proteomics sequencing.

**Additional file 2: Supplementary Table S2.** Identifed proteins associated with HDAC6 were analyzed by mass spectrometry.

**Additional file 3. **

## Data Availability

All data generated or analyzed during this study are included in this published article (and its supplementary information files).

## Abbreviations

ANOVA: Analysis of variance
HATs: Histone acetyltransferases
HCC: Hepatocellular carcinoma
HDAC2: Histone deacetylase 2
HDAC6: Histone deacetylase 6
HDACs: Histone deacetylases
HPLC: Liquid chromatography
IP: Immunoprecipitation
LC–MS/MS: Liquid chromatography coupled with tandem mass spectrometry
LukS-PV: S component of Panton-Valetine leucocidin
MMP2: Matrix metallopeptidase 2
MMP9: Matrix metallopeptidase 9
MS/MS: Mass spectrometry
NSI: Nano spray ionization
PCR: Polymerase chain reaction
PVL: Panton-Valetine leucocidin
RT-PCR: Reverse transcription-polymerase Chain reaction
SDS-PAGE: Sodium dodecyl sulfate polyacrylamide gel electrophoresis
TEAB: Tetraethyl ammonium bromide
UPLC: Ultraperformance liquid chromatography
