# Supplementary material for: LukS-PV inhibits hepatocellular carcinoma cells migration by downregulating HDAC6 expression
Source: BMC Cancer. 2022 Jun 8;22:630. doi: 10.1186/s12885-022-09680-4 (PMC9175482; doi:10.1186/s12885-022-09680-4)
Supplement: Supplementary file 3 — Additional file 3. [file 12885_2022_9680_MOESM3_ESM.pdf]

Figure 1C

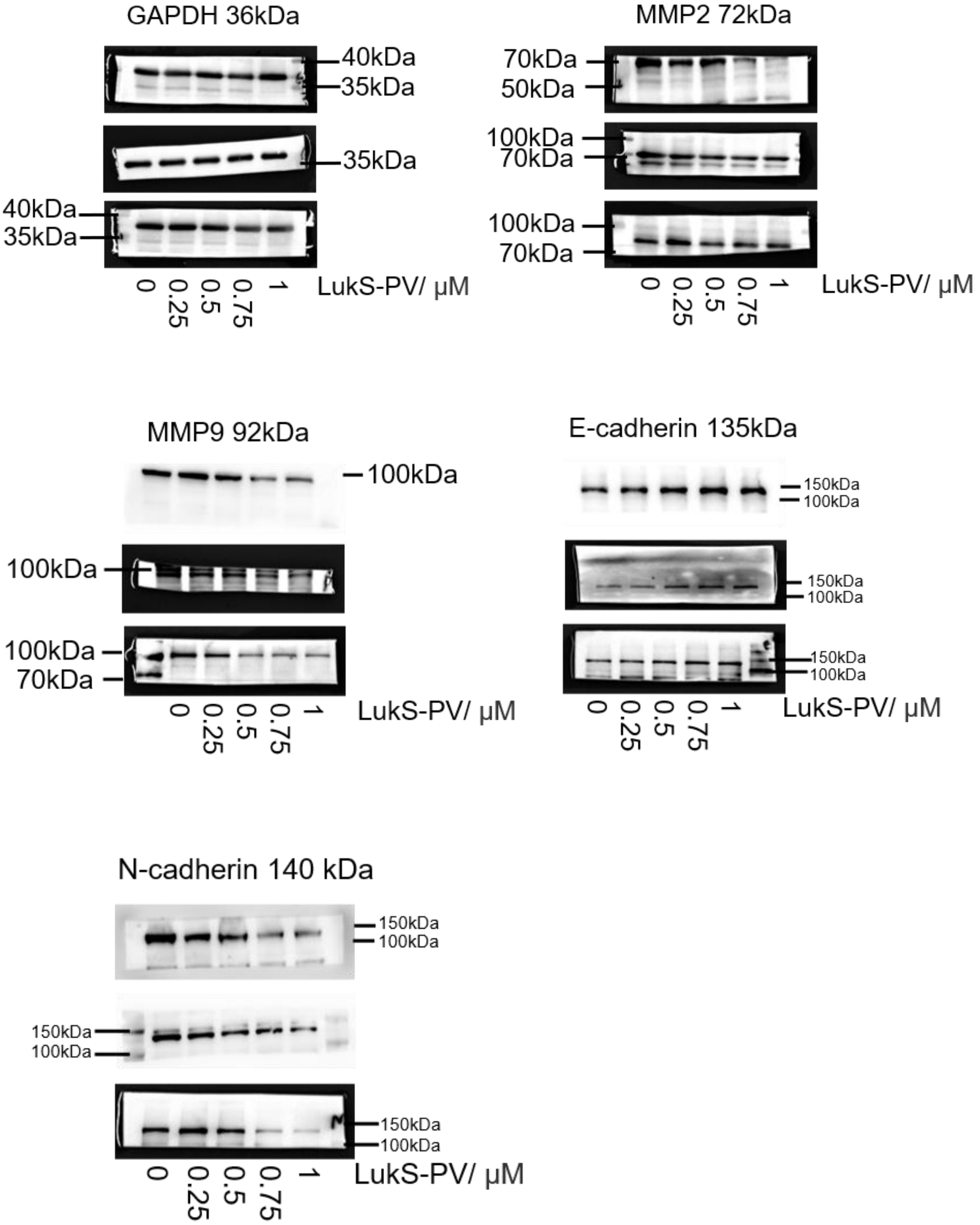

GAPDH 36kDa
40kDa
35kDa
35kDa
40kDa
35kDa
0
0.25
0.5
0.75
1
LukS-PV/ μM
MMP2 72kDa
70kDa
50kDa
100kDa
70kDa
100kDa
70kDa
0
0.25
0.5
0.75
1
LukS-PV/ μM
MMP9 92kDa
100kDa
100kDa
100kDa
70kDa
0
0.25
0.5
0.75
1
LukS-PV/ μM
E-cadherin 135kDa
150kDa
100kDa
150kDa
100kDa
150kDa
100kDa
0
0.25
0.5
0.75
1
LukS-PV/ μM
N-cadherin 140 kDa
150kDa
100kDa
150kDa
100kDa
150kDa
100kDa
0
0.25
0.5
0.75
1
LukS-PV/ μM

Figure 1D

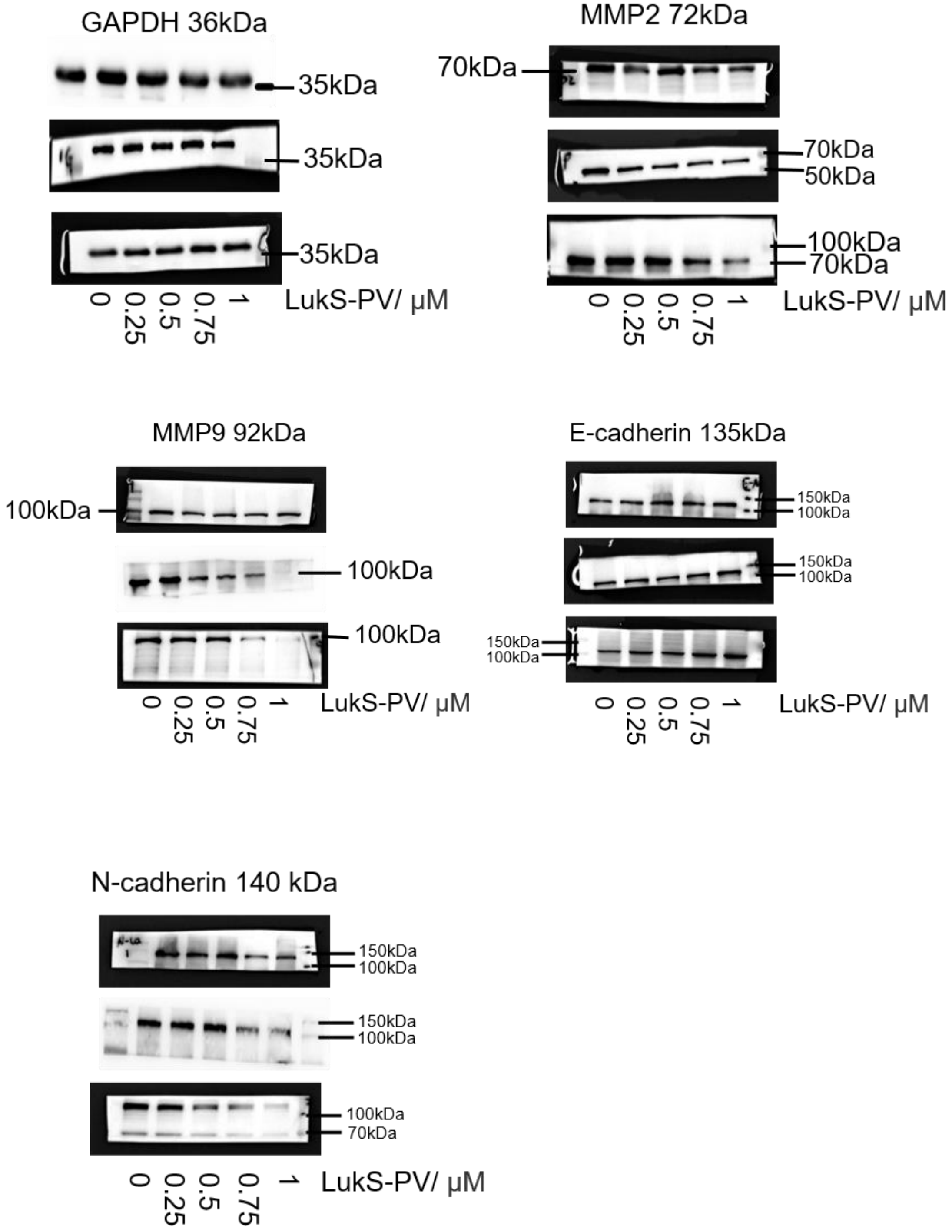

GAPDH 36kDa
35kDa
35kDa
35kDa
0
0.25
0.5
0.75
1
LukS-PV/ μM
MMP2 72kDa
70kDa
70kDa
50kDa
100kDa
70kDa
LukS-PV/ μM
MMP9 92kDa
100kDa
100kDa
100kDa
LukS-PV/ μM
E-cadherin 135kDa
150kDa
100kDa
LukS-PV/ μM
N-cadherin 140 kDa
150kDa
100kDa
70kDa
LukS-PV/ μM

Figure 2B

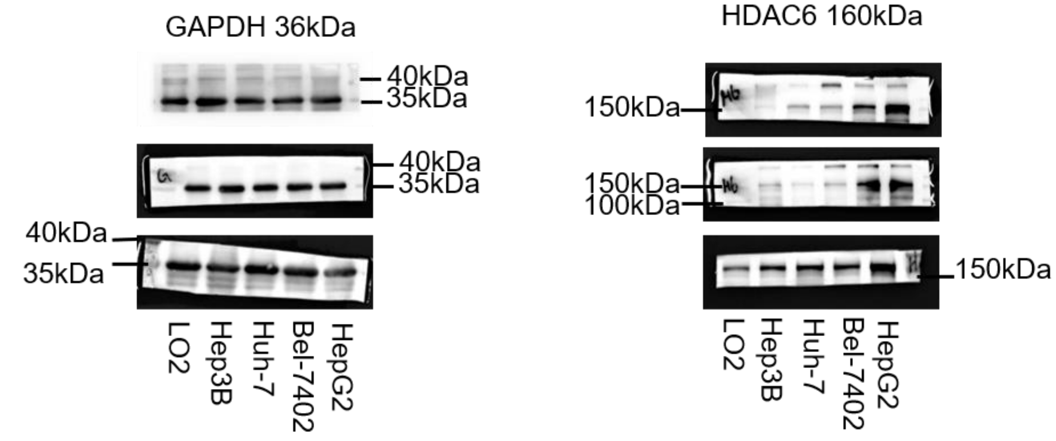

Figure 2C

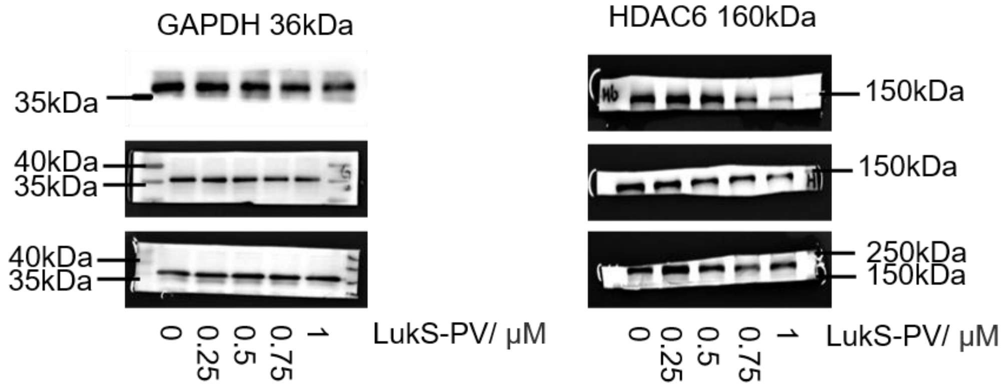

Figure 2D

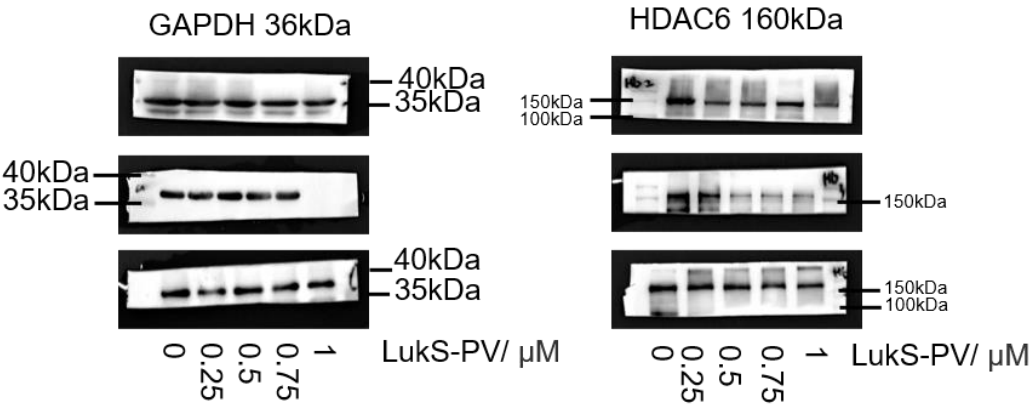

Figure 3C

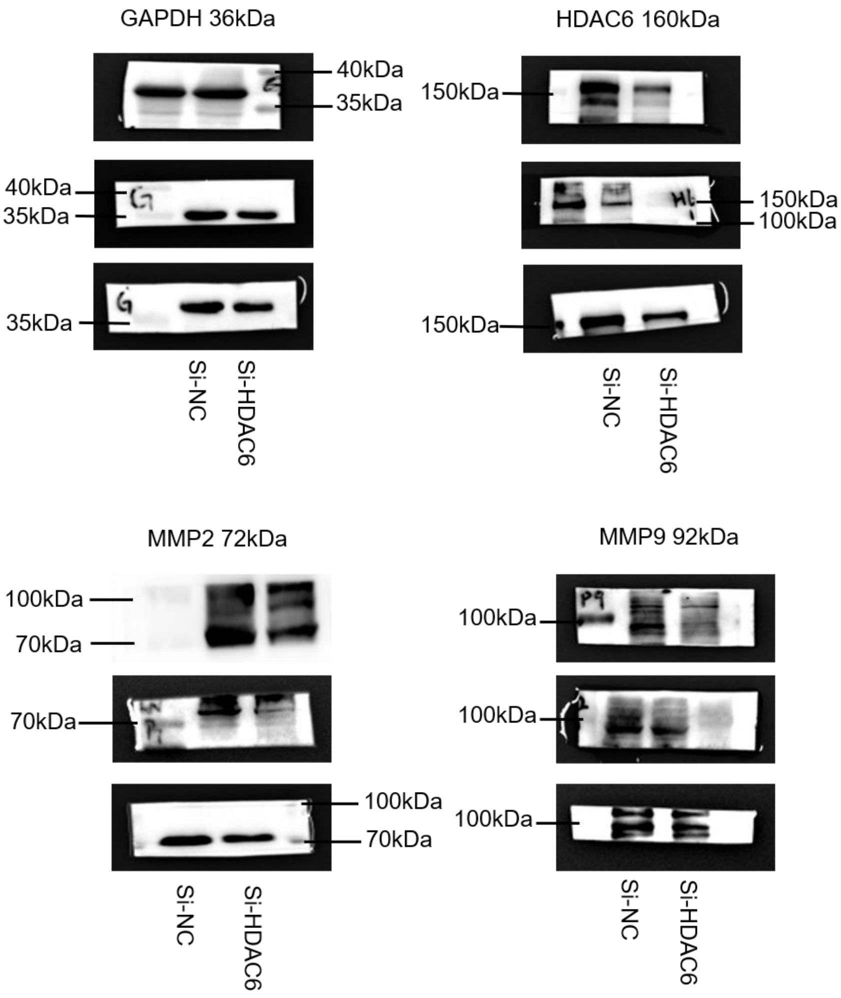

Figure 3D

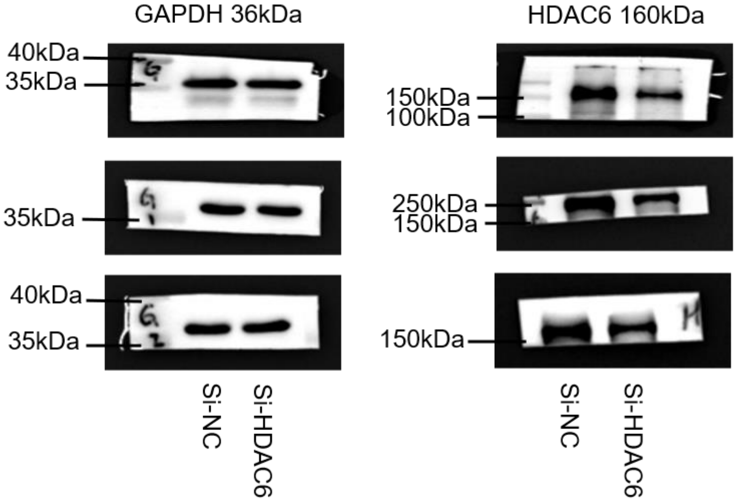

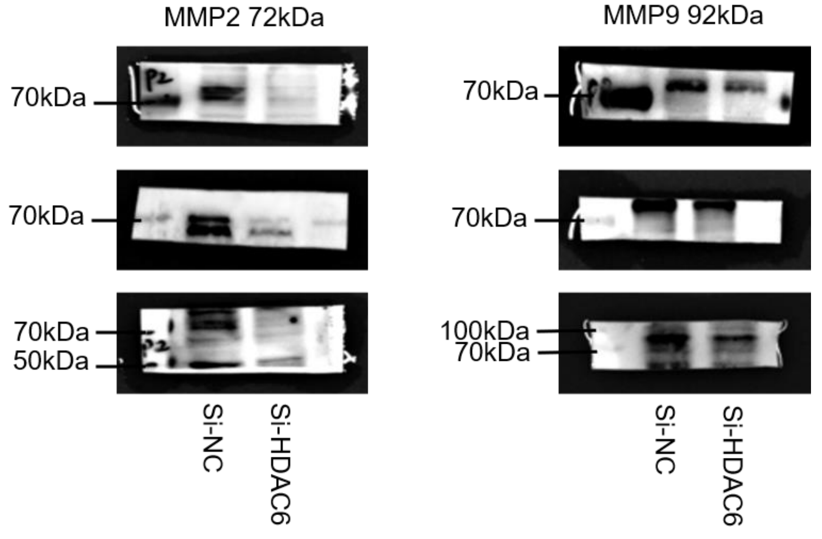
MMP2 72kDa
MMP9 92kDa
70kDa
70kDa
70kDa
50kDa
70kDa
70kDa
100kDa
70kDa
Si-NC
Si-HDAC6
Si-NC
Si-HDAC6

Figure 4C

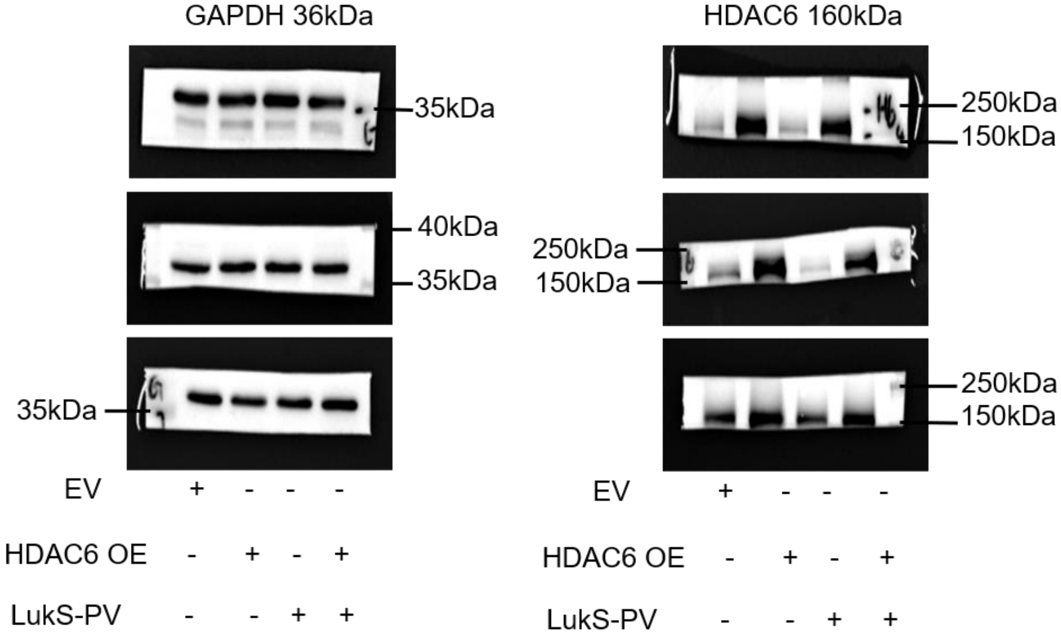
GAPDH 36kDa
HDAC6 160kDa
35kDa
40kDa
35kDa
35kDa
250kDa
150kDa
250kDa
150kDa
250kDa
150kDa
EV + - - -
HDAC6 OE - + - +
LukS-PV - - + +
EV + - - -
HDAC6 OE - + - +
LukS-PV - - + +

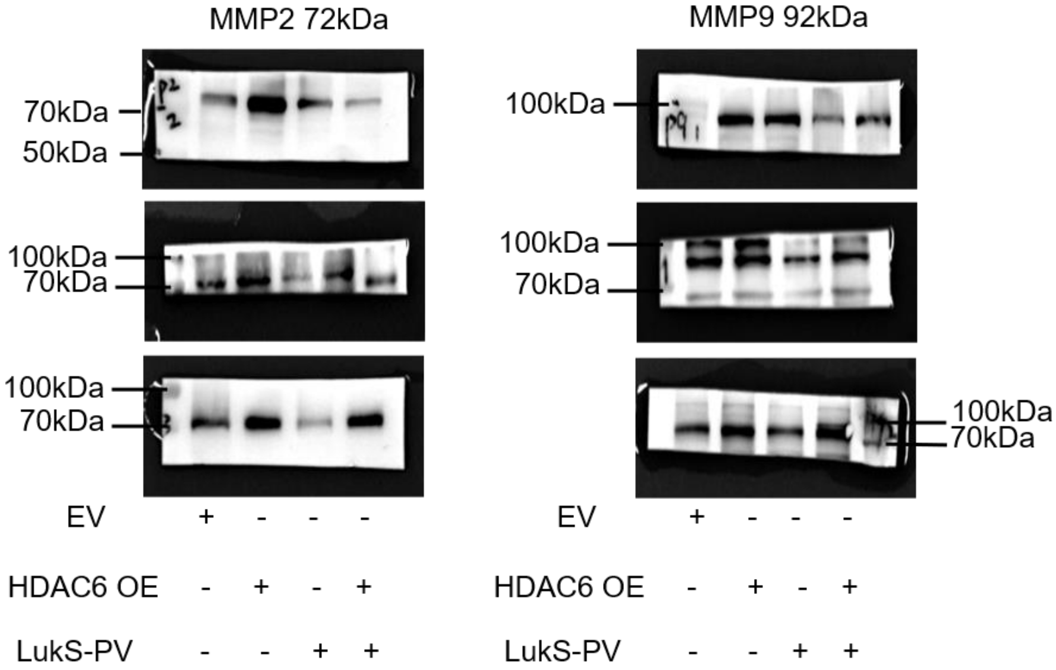
MMP2 72kDa
70kDa
50kDa
100kDa
70kDa
100kDa
70kDa
EV + - - -
HDAC6 OE - + - +
LukS-PV - - + +
MMP9 92kDa
100kDa
100kDa
70kDa
100kDa
70kDa
EV + - - -
HDAC6 OE - + - +
LukS-PV - - + +

Figure 4D

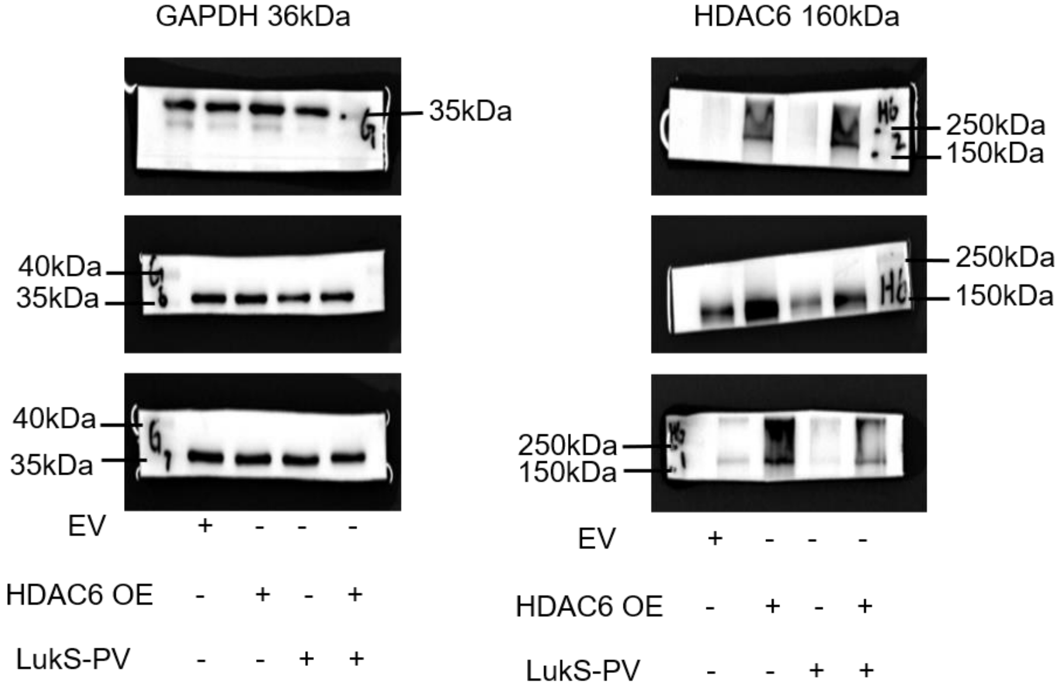
GAPDH 36kDa
35kDa
40kDa
35kDa
40kDa
35kDa
EV + - - -
HDAC6 OE - + - +
LukS-PV - - + +
HDAC6 160kDa
250kDa
150kDa
250kDa
150kDa
250kDa
150kDa
EV + - - -
HDAC6 OE - + - +
LukS-PV - - + +

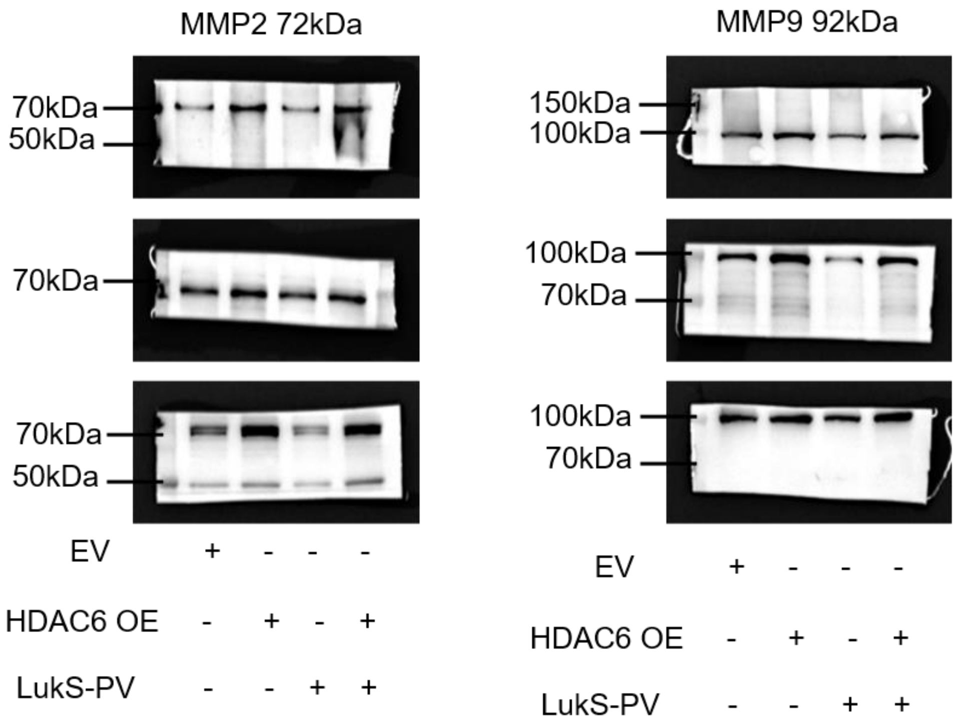
MMP2 72kDa
MMP9 92kDa
70kDa
50kDa
70kDa
70kDa
50kDa
150kDa
100kDa
100kDa
70kDa
100kDa
70kDa
EV + - - -
HDAC6 OE - + - +
LukS-PV - - + +
EV + - - -
HDAC6 OE - + - +
LukS-PV - - + +

Figure 5A

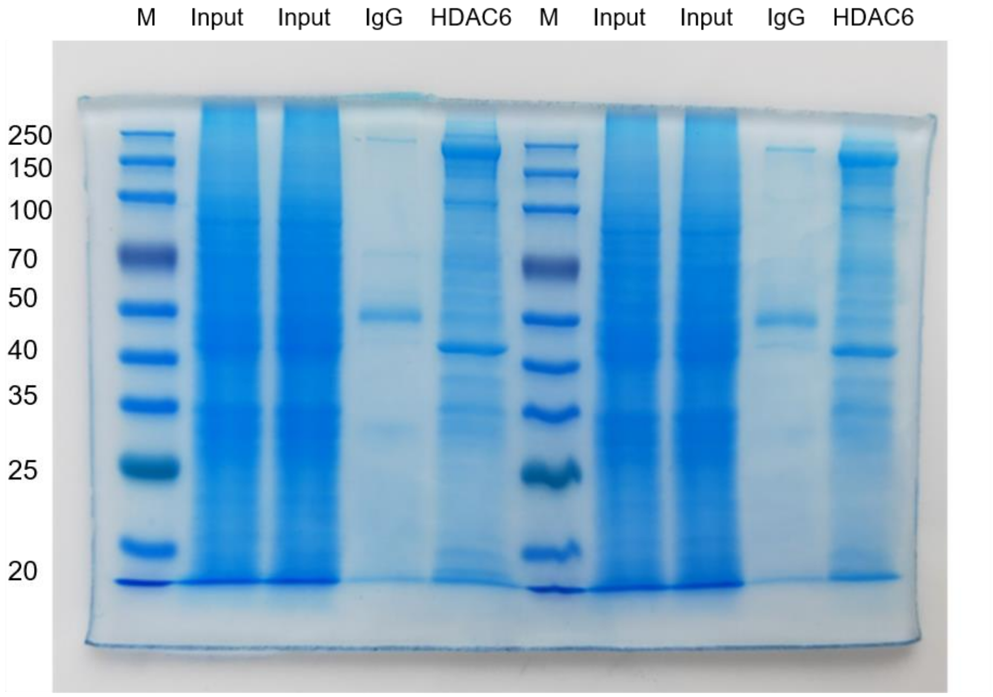
M Input Input IgG HDAC6 M Input Input IgG HDAC6
250
150
100
70
50
40
35
25
20

Figure 5B

Hep3B

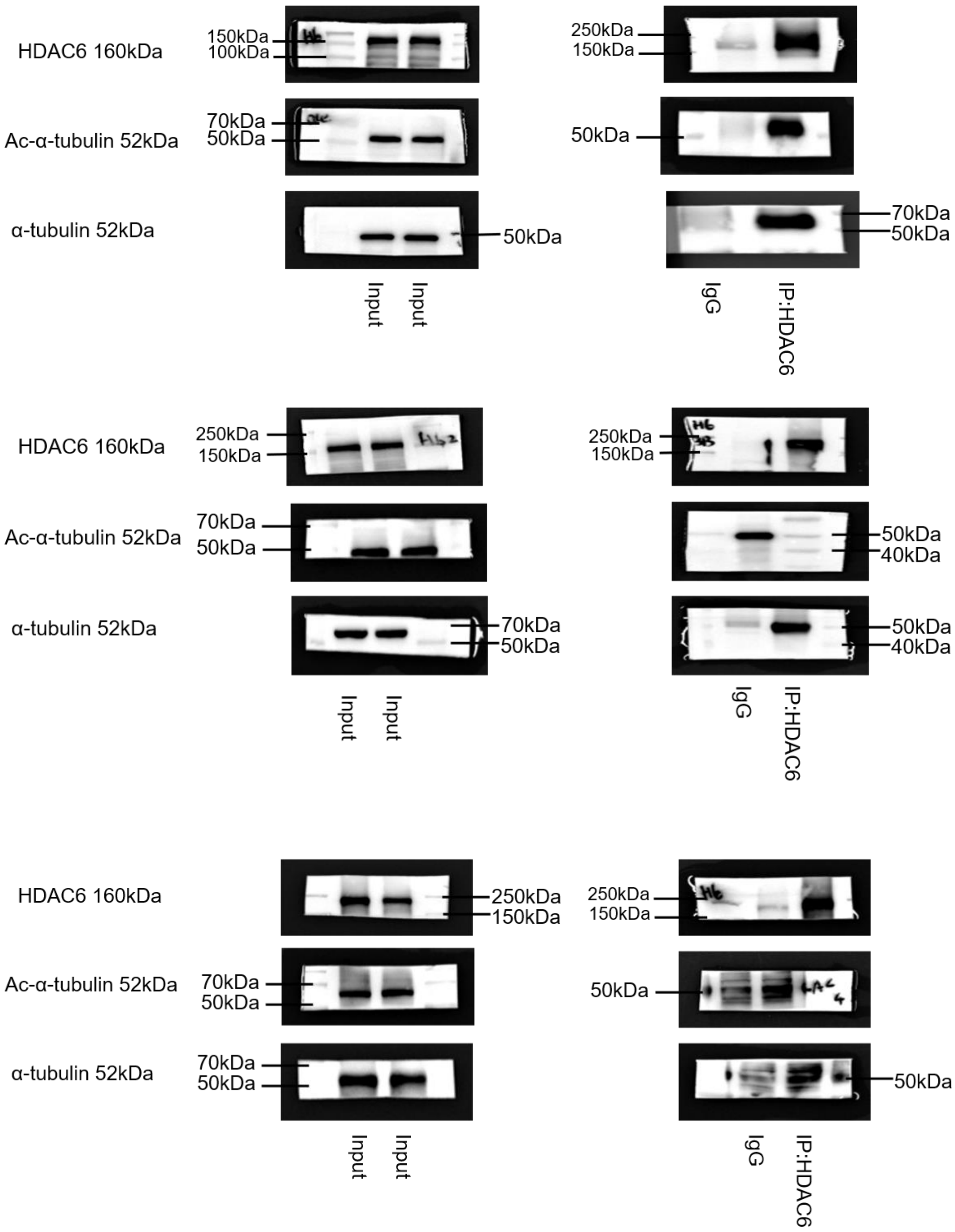

Figure 5B

Huh-7

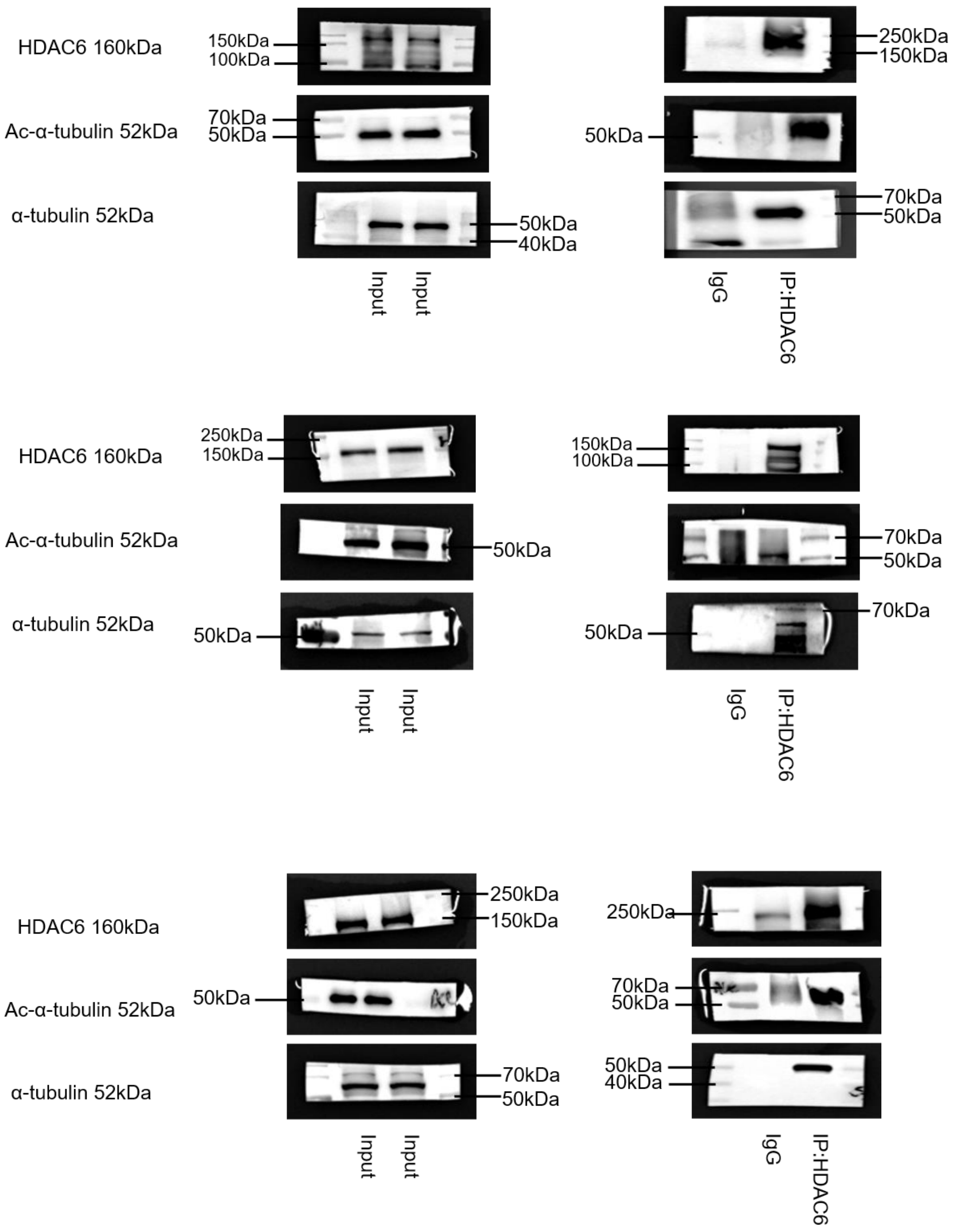

Figure 5C

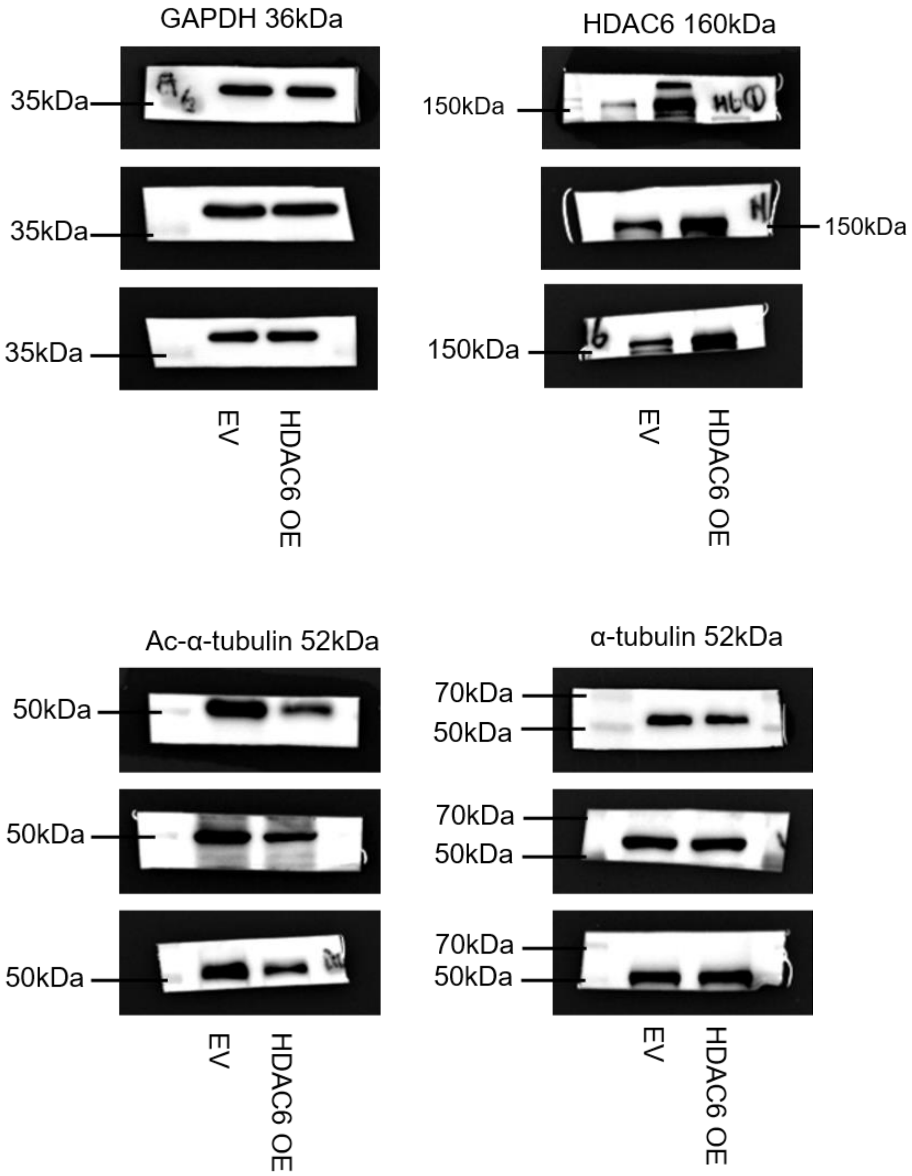
GAPDH 36kDa
35kDa
35kDa
35kDa
HDAC6 160kDa
150kDa
150kDa
150kDa
EV
HDAC6 OE
EV
HDAC6 OE
Ac-α-tubulin 52kDa
50kDa
50kDa
50kDa
α-tubulin 52kDa
70kDa
50kDa
70kDa
50kDa
70kDa
50kDa
EV
HDAC6 OE
EV
HDAC6 OE

Figure 5D

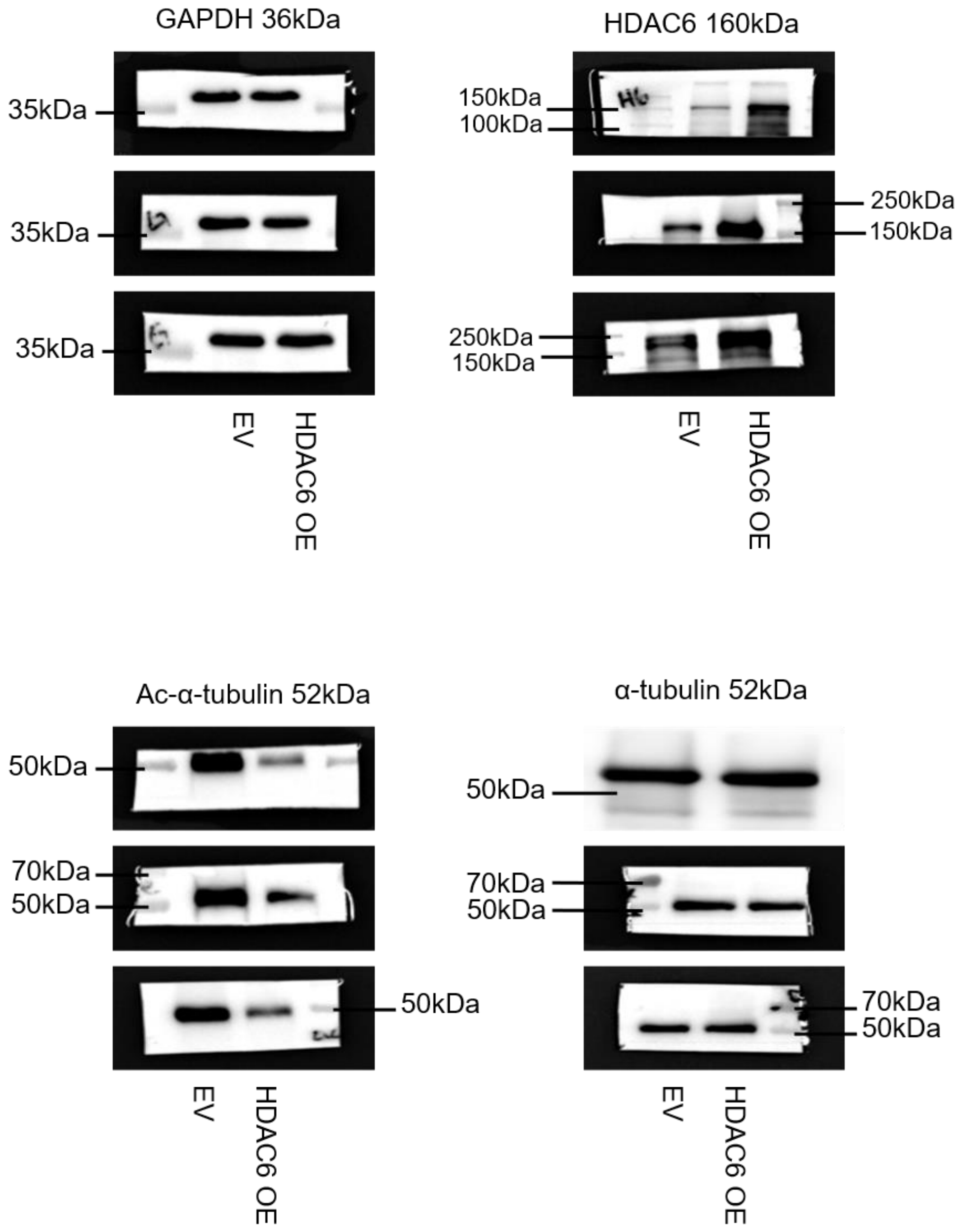
GAPDH 36kDa
35kDa
35kDa
35kDa
HDAC6 160kDa
150kDa
100kDa
250kDa
150kDa
250kDa
150kDa
EV
HDAC6 OE
EV
HDAC6 OE
Ac-α-tubulin 52kDa
50kDa
70kDa
50kDa
50kDa
α-tubulin 52kDa
50kDa
70kDa
50kDa
70kDa
50kDa
EV
HDAC6 OE
EV
HDAC6 OE

Figure 5E

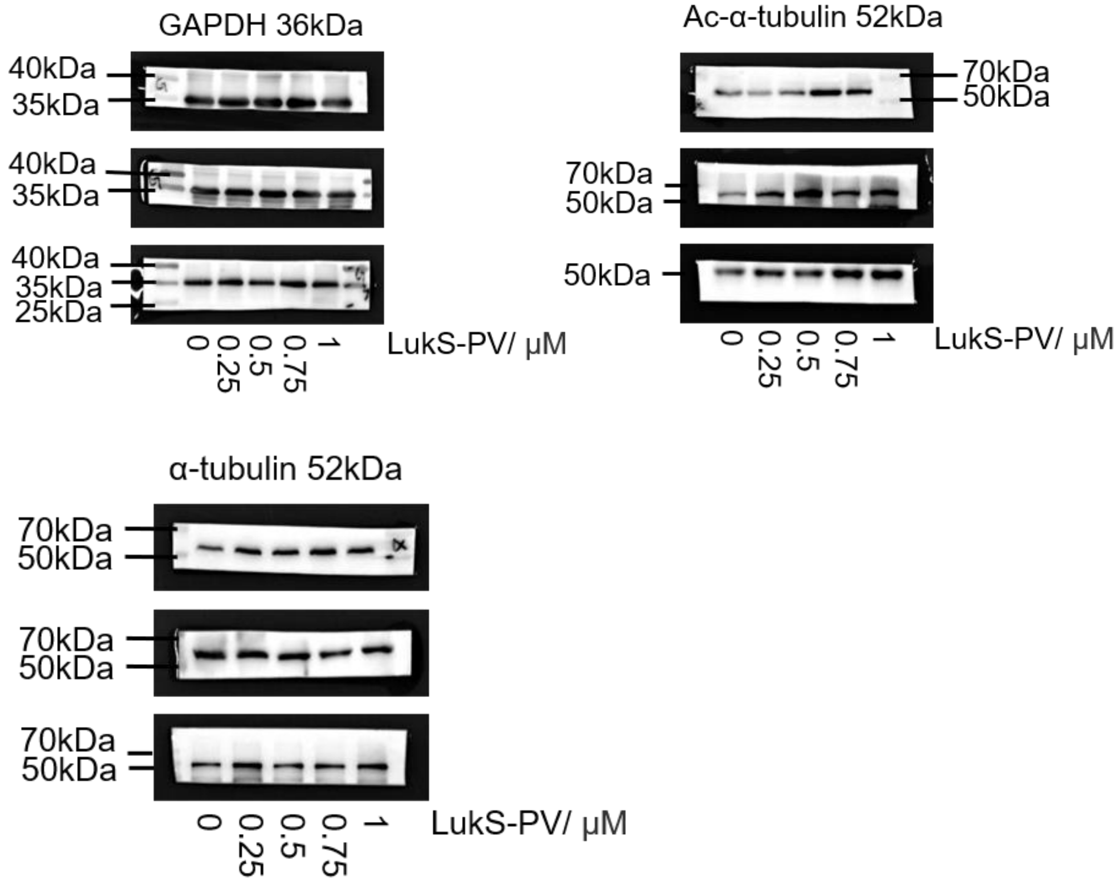

Figure 5F

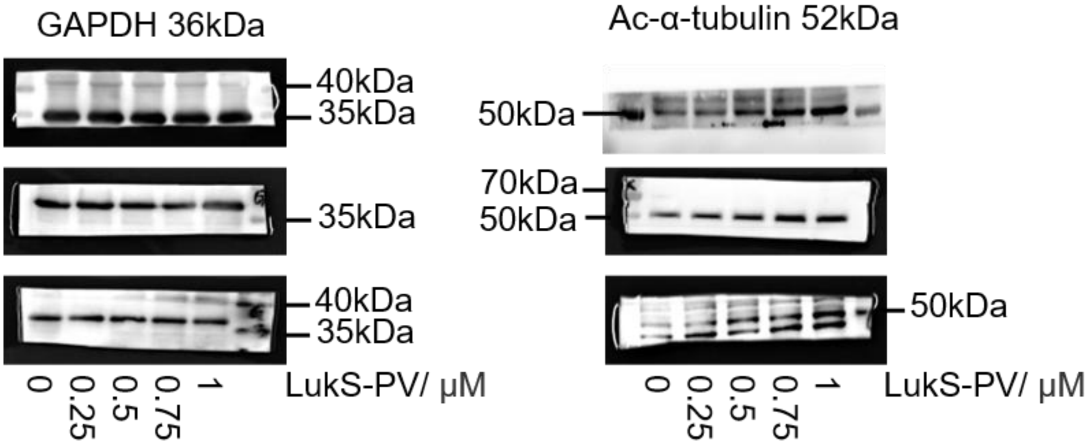

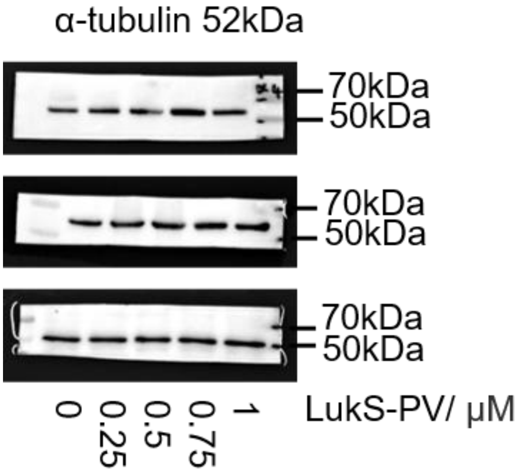
α-tubulin 52kDa
70kDa
50kDa
70kDa
50kDa
70kDa
50kDa
0
0.25
0.5
0.75
1
LukS-PV/ μM

Figure 6C

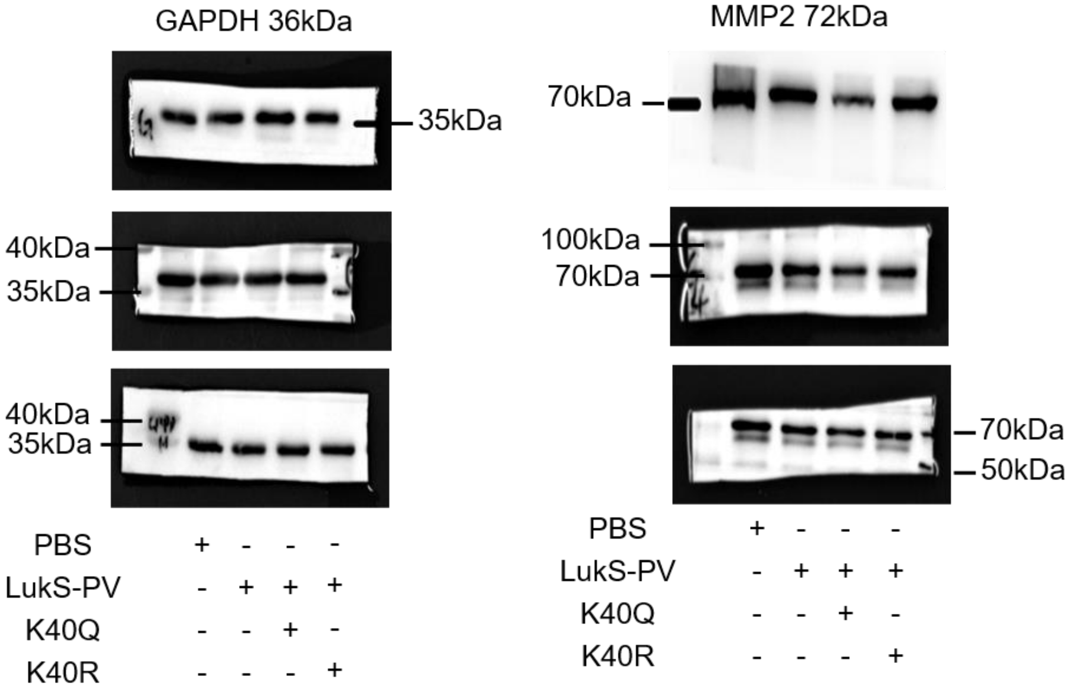
GAPDH 36kDa
35kDa
40kDa
35kDa
40kDa
35kDa
PBS + - - -
LukS-PV - + + +
K40Q - - + -
K40R - - - +
MMP2 72kDa
70kDa
100kDa
70kDa
70kDa
50kDa
PBS + - - -
LukS-PV - + + +
K40Q - - + -
K40R - - - +

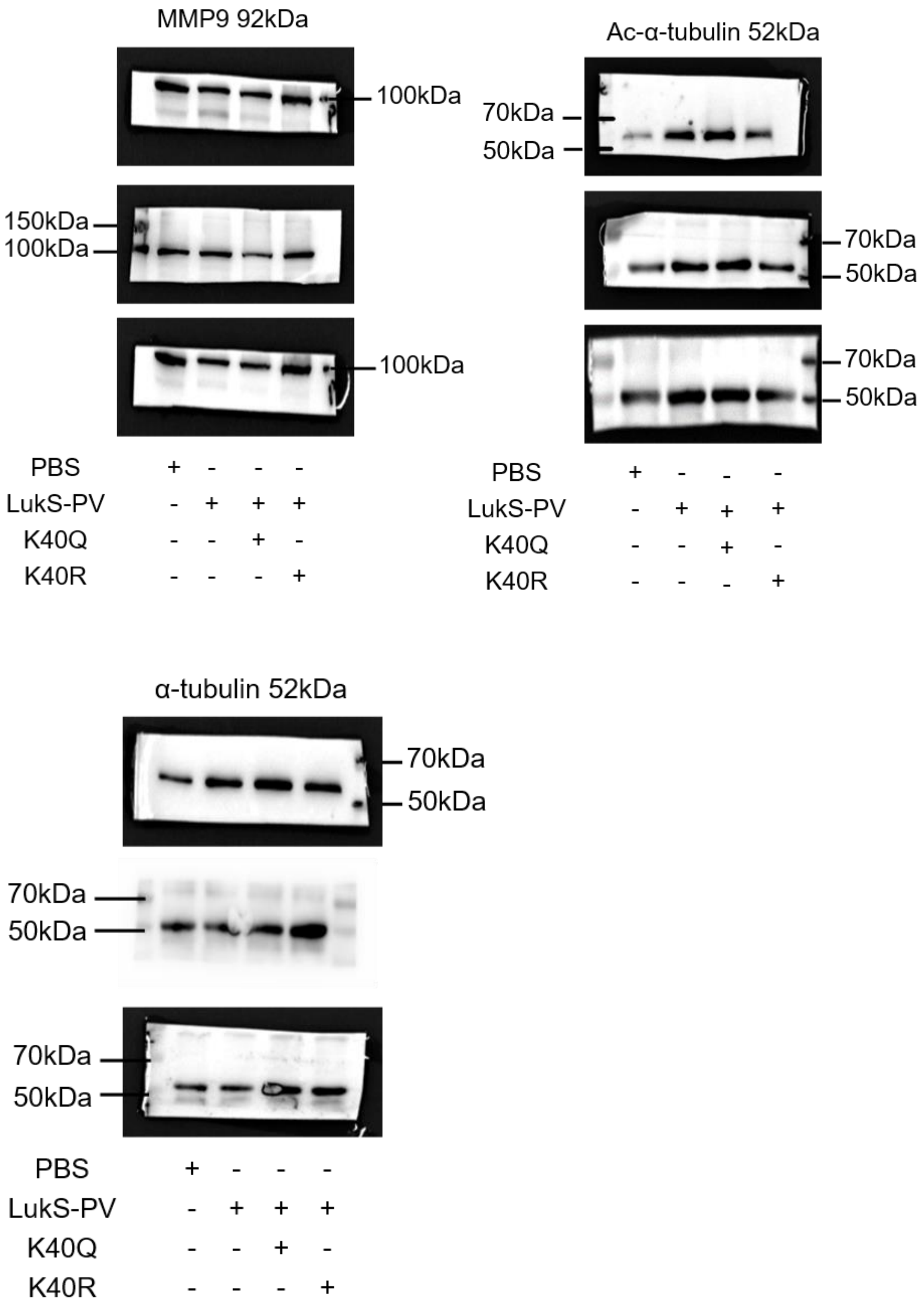
MMP9 92kDa
100kDa
150kDa
100kDa
100kDa
PBS + - - -
LukS-PV - + + +
K40Q - - + -
K40R - - - +
Ac-α-tubulin 52kDa
70kDa
50kDa
70kDa
50kDa
70kDa
50kDa
PBS + - - -
LukS-PV - + + +
K40Q - - + -
K40R - - - +
α-tubulin 52kDa
70kDa
50kDa
70kDa
50kDa
70kDa
50kDa
PBS + - - -
LukS-PV - + + +
K40Q - - + -
K40R - - - +

Figure 6D

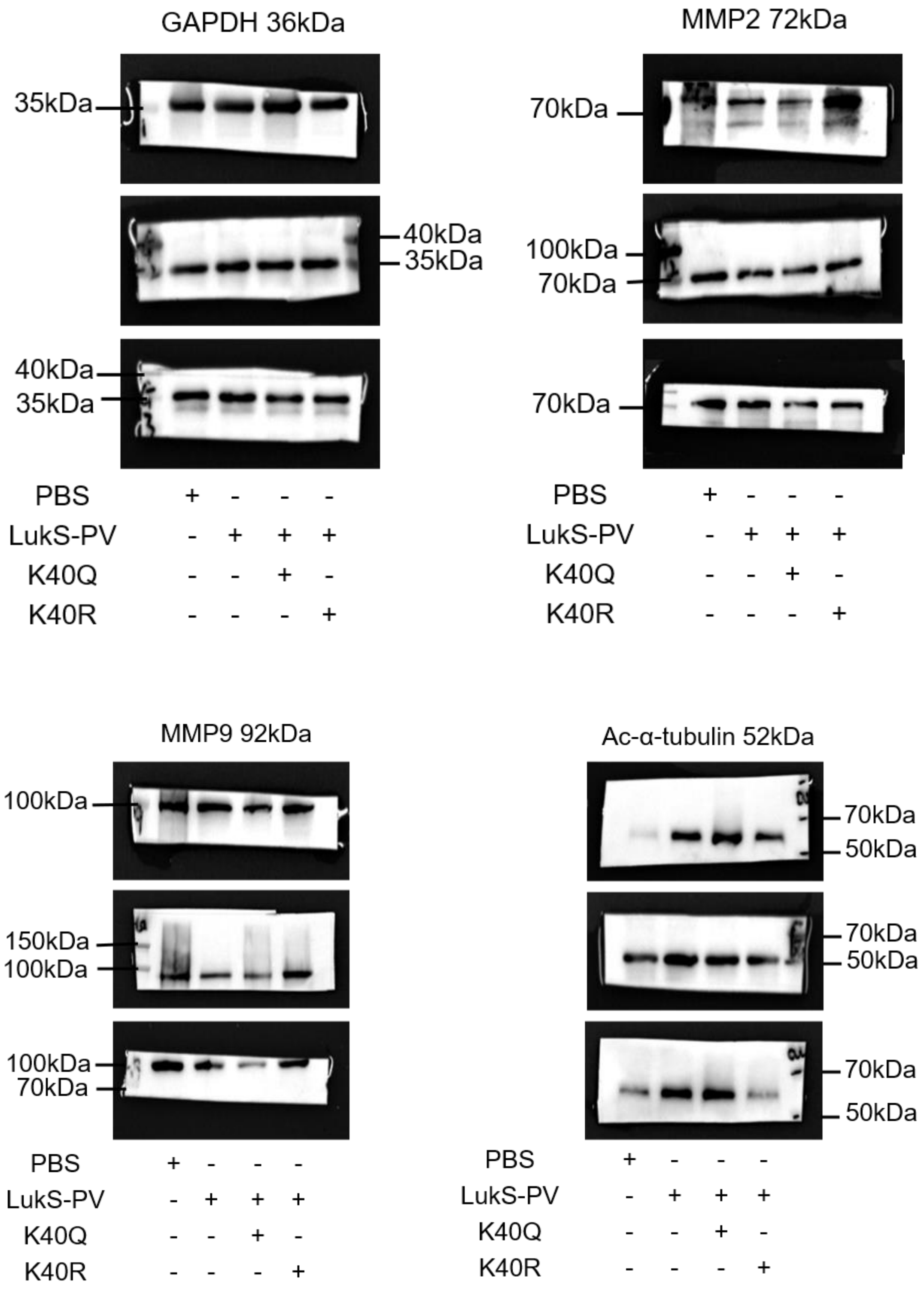

GAPDH 36kDa
35kDa
40kDa
35kDa
40kDa
35kDa
PBS + - - -
LukS-PV - + + +
K40Q - - + -
K40R - - - +
MMP2 72kDa
70kDa
100kDa
70kDa
70kDa
PBS + - - -
LukS-PV - + + +
K40Q - - + -
K40R - - - +
MMP9 92kDa
100kDa
150kDa
100kDa
100kDa
70kDa
PBS + - - -
LukS-PV - + + +
K40Q - - + -
K40R - - - +
Ac-α-tubulin 52kDa
70kDa
50kDa
70kDa
50kDa
70kDa
50kDa
PBS + - - -
LukS-PV - + + +
K40Q - - + -
K40R - - - +

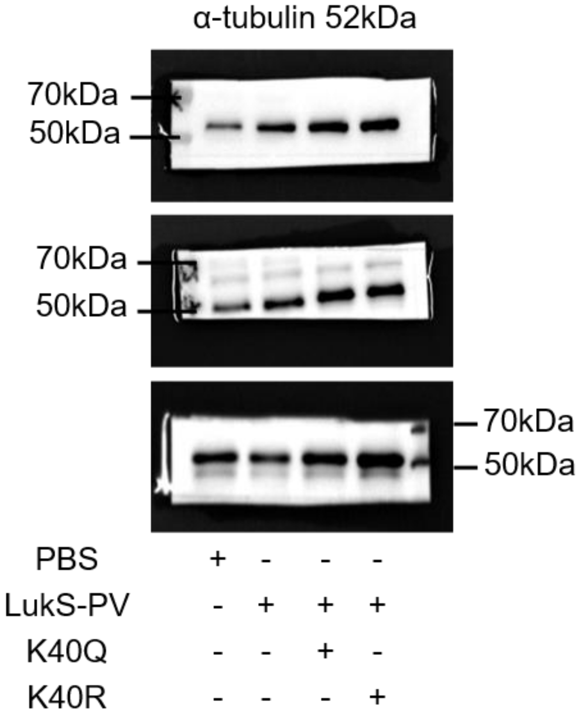
α-tubulin 52kDa
70kDa
50kDa
70kDa
50kDa
70kDa
50kDa
PBS + - - -
LukS-PV - + + +
K40Q - - + -
K40R - - - +

Figure 6G

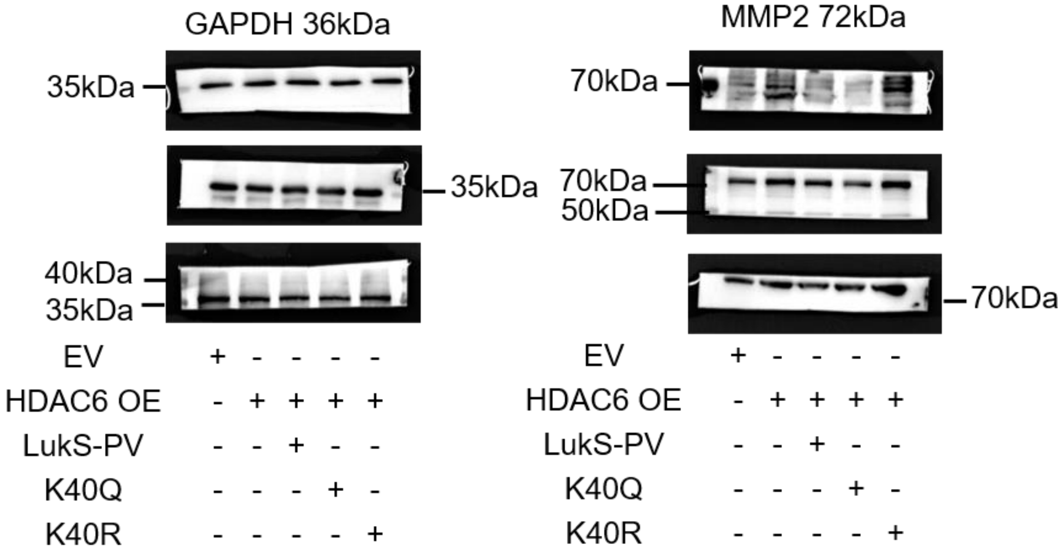
GAPDH 36kDa
35kDa
35kDa
40kDa
35kDa
EV + - - - -
HDAC6 OE - + + + +
LukS-PV - - + - -
K40Q - - - + -
K40R - - - - +
MMP2 72kDa
70kDa
70kDa
50kDa
70kDa
EV + - - - -
HDAC6 OE - + + + +
LukS-PV - - + - -
K40Q - - - + -
K40R - - - - +

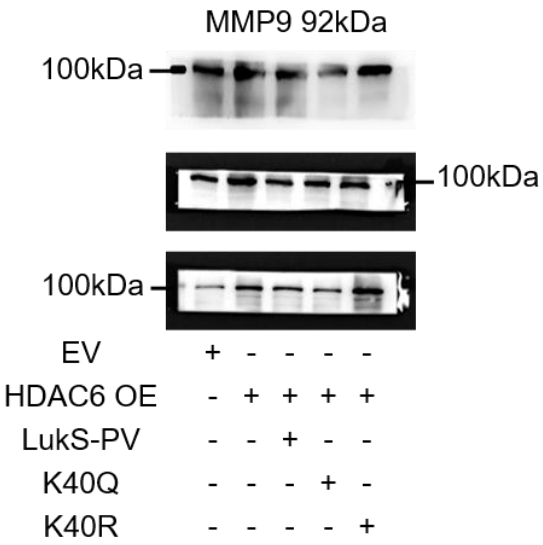
MMP9 92kDa
100kDa
100kDa
100kDa
EV + - - - -
HDAC6 OE - + + + +
LukS-PV - - + - -
K40Q - - - + -
K40R - - - - +

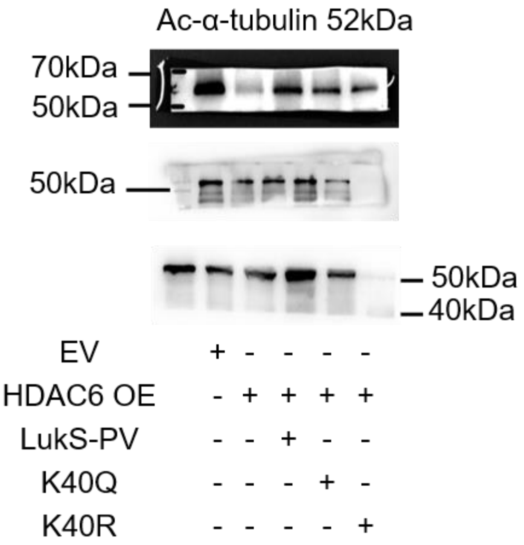
Ac-α-tubulin 52kDa
70kDa
50kDa
50kDa
50kDa
40kDa
EV + - - - -
HDAC6 OE - + + + +
LukS-PV - - + - -
K40Q - - - + -
K40R - - - - +

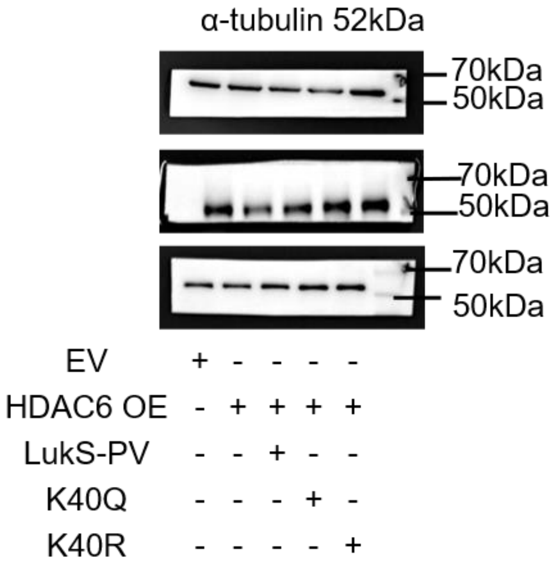
α-tubulin 52kDa
70kDa
50kDa
70kDa
50kDa
70kDa
50kDa
EV + - - - -
HDAC6 OE - + + + +
LukS-PV - - + - -
K40Q - - - + -
K40R - - - - +

Figure 6H

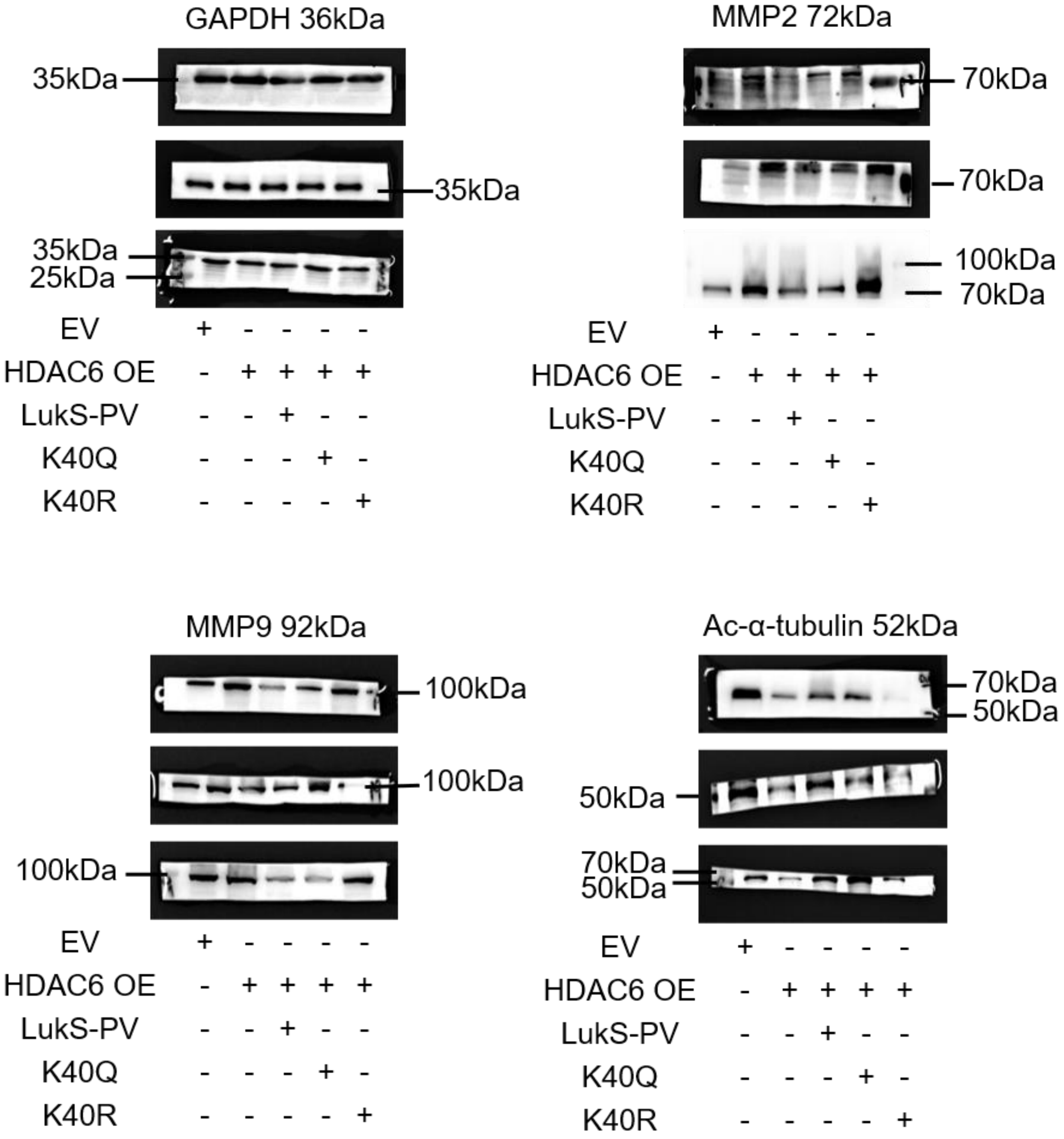
GAPDH 36kDa
35kDa
35kDa
35kDa
25kDa
MMP2 72kDa
70kDa
70kDa
100kDa
70kDa
EV + - - - -
HDAC6 OE - + + + +
LukS-PV - - + - -
K40Q - - - + -
K40R - - - - +
MMP9 92kDa
100kDa
100kDa
100kDa
Ac-α-tubulin 52kDa
70kDa
50kDa
50kDa
70kDa
50kDa
EV + - - - -
HDAC6 OE - + + + +
LukS-PV - - + - -
K40Q - - - + -
K40R - - - - +

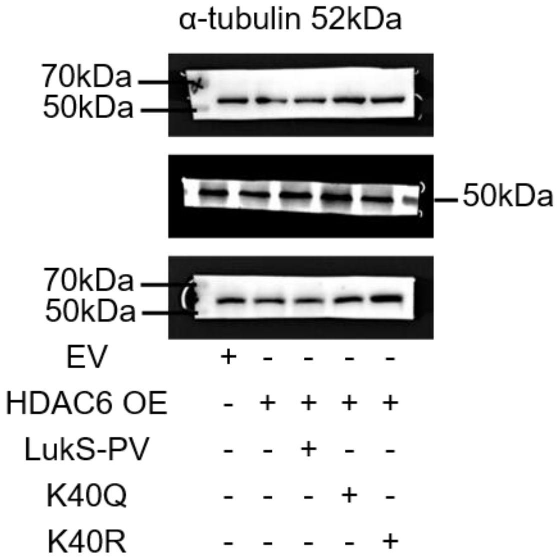
α-tubulin 52kDa
70kDa
50kDa
50kDa
70kDa
50kDa
EV + - - - -
HDAC6 OE - + + + +
LukS-PV - - + - -
K40Q - - - + -
K40R - - - - +
